# Analytical applications of molecularly imprinted polymers: a personal view

**DOI:** 10.1007/s00216-026-06800-y

**Published:** 2026-09-21

**Authors:** Aysu Yarman, Aysel Oktay, Emine Sezer, Sinan Akgol, Anne Ettner, Zeynep Sabahat Yunt Ghanbari, Cem Bulent Ustundag, Frieder W. Scheller

**Affiliations:** 1 Molecular Biotechnology, Faculty of Science, Turkish-German University, Istanbul, 34820 Turkey; 2 Department of Bioengineering, Faculty of Chemical and Metallurgical Engineering, Yildiz Technical University, Istanbul, 34220 Turkey; 3 Biorege Polymeric Nanosystems & AI Assisted Applications Laboratory, Ege University, Bornova, Izmir, 35100 Turkey; 4 Department of Computer Engineering, Faculty of Computer and Information Sciences, Ege University, Bornova, Izmir, 35100 Turkey; 5 Department of Biochemistry, Faculty of Science, Ege University, Bornova, Izmir, 35100 Turkey; 6 Anne Ettner Pharma Consulting/Wollbach, 79400 Kandern, Germany; 7 Institute of Biochemistry and Biology, University of Potsdam, Karl-Liebknecht-Strasse 24-25, 14476 Potsdam, Germany

**Keywords:** Molecularly imprinted polymers, Biomimetic sensors, Epitope imprinting, Artificial intelligence (AI), Machine learning

## Abstract

Molecularly imprinted polymers (MIPs) are biomimetic counterparts of biomacromolecules that are synthesized by templating the target within a polymeric network. While the affinity of MIPs can compete with that of antibodies, the specific activity of natural enzymes remains superior to that of catalytically active MIPs. Although the mechanisms underlying the generation of antibodies and MIPs are fundamentally different, important principles have been transferred from biology to artificial binders. Templating a fragment of the target instead of the entire molecule or particle simplifies MIP synthesis and reduces costs (epitope imprinting), while hierarchical imprinting improves template orientation and generates more homogeneous binding sites than conventional random imprinting. The generation of anti-idiotypic MIPs by double imprinting follows the concept of anti-idiotypic antibodies, while post-imprinting modifications mimic post-translational modifications. Beyond classical terminal epitopes, recent approaches increasingly utilize internal epitopes, affinity tags, labels, carbohydrates, and surface motifs of viruses and cells as alternative imprinting targets. Recent advances in analytical MIP systems increasingly rely on the integration of nanomaterials to improve signal generation, mass transport, and target accessibility. In this context, nanoparticles, carbon-based nanomaterials, MXenes, and metal-organic frameworks (MOFs) have enabled the development of more efficient and functionally integrated sensing platforms. In parallel, computational rational design, artificial intelligence, and machine learning–assisted signal deconvolution are emerging as powerful tools for optimizing monomer selection, interpreting complex sensor outputs, and enabling multiplex analysis using cross-reactive MIP sensor arrays. Despite clear advantages such as higher stability, lower cost, and applicability to toxic or weakly immunogenic substances, the commercial success of MIPs is still restricted by challenges related to selectivity, reproducibility, and translation into robust real-world analytical platforms. This review discusses the evolution of analytical MIPs from classical molecular recognition materials toward increasingly intelligent biomimetic sensing systems.

**Supplementary Information:**

The online version contains supplementary material available at https://doi.org/10.1007/s00216-026-06800-y.

## Introduction

The history of analytical science can be interpreted as a continuous attempt to translate invisible molecular events into measurable physical signals. From the earliest biochemical assays to modern biosensors, this transformation has relied on the coupling of recognition and transduction—a principle that has remained unchanged for nearly a century. The introduction of the optical test by Otto Warburg in 1936 marked one of the first systematic efforts to monitor biological activity via physicochemical readouts, establishing a paradigm in which molecular processes could be quantified indirectly through signal generation. This conceptual framework laid the foundation for subsequent developments, including enzyme electrodes introduced by Leland C. Clark in the 1960s, which enabled the electrochemical monitoring of biochemical reactions [1].

Parallel to these developments, the idea of encoding molecular information into synthetic materials emerged with the pioneering work of Mikhail V. Polyakov in 1931, who demonstrated that polymer matrices could retain a form of “molecular memory.” However, it was not until the contributions of Günter Wulff and Klaus Mosbach in the 1970s and 1980 s that molecular imprinting evolved into a reproducible and widely applicable strategy [2, 3]. These developments paralleled the emergence of monoclonal antibodies by César Milstein and Georges Köhler, highlighting two fundamentally different approaches to molecular recognition: biological evolution versus synthetic design [4].

The subsequent decades, as reflected in Table 1, reveal not merely a sequence of technological innovations but an increasing convergence of biological and synthetic recognition systems. The advent of nucleic acid–based recognition elements, particularly aptamers generated by systematic evolution of ligands by exponential enrichment (SELEX) [5, 6], introduced a programmable alternative to antibodies, while DNA chips and surface plasmon resonance systems enabled multiplexed and label-free detection [7, 8]. In this context, molecularly imprinted polymers (MIPs) occupy a unique position: they are neither biological nor purely artificial in the traditional sense but rather represent a hybrid conceptual class in which chemical synthesis is used to emulate biological function.

**Table 1:**
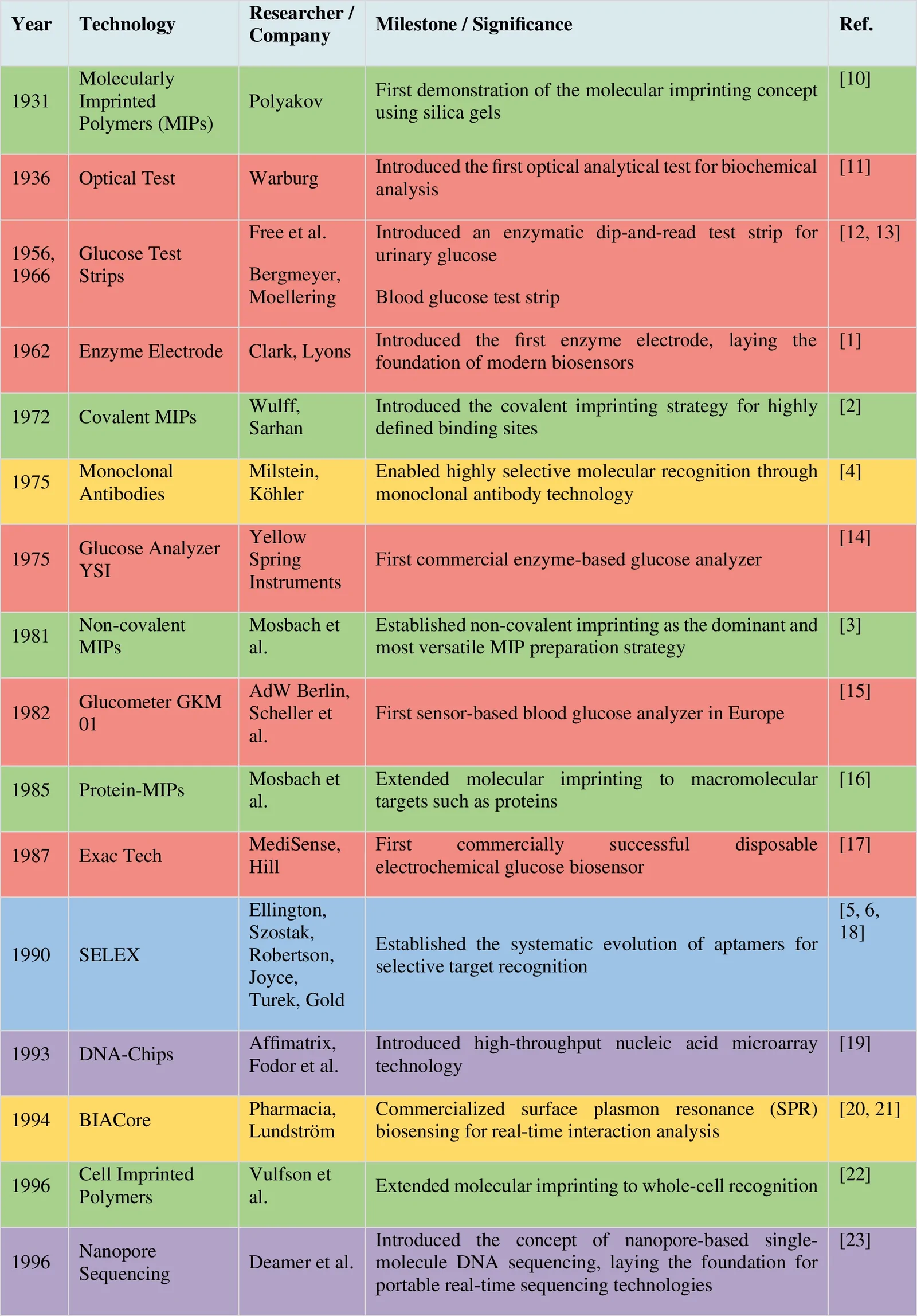

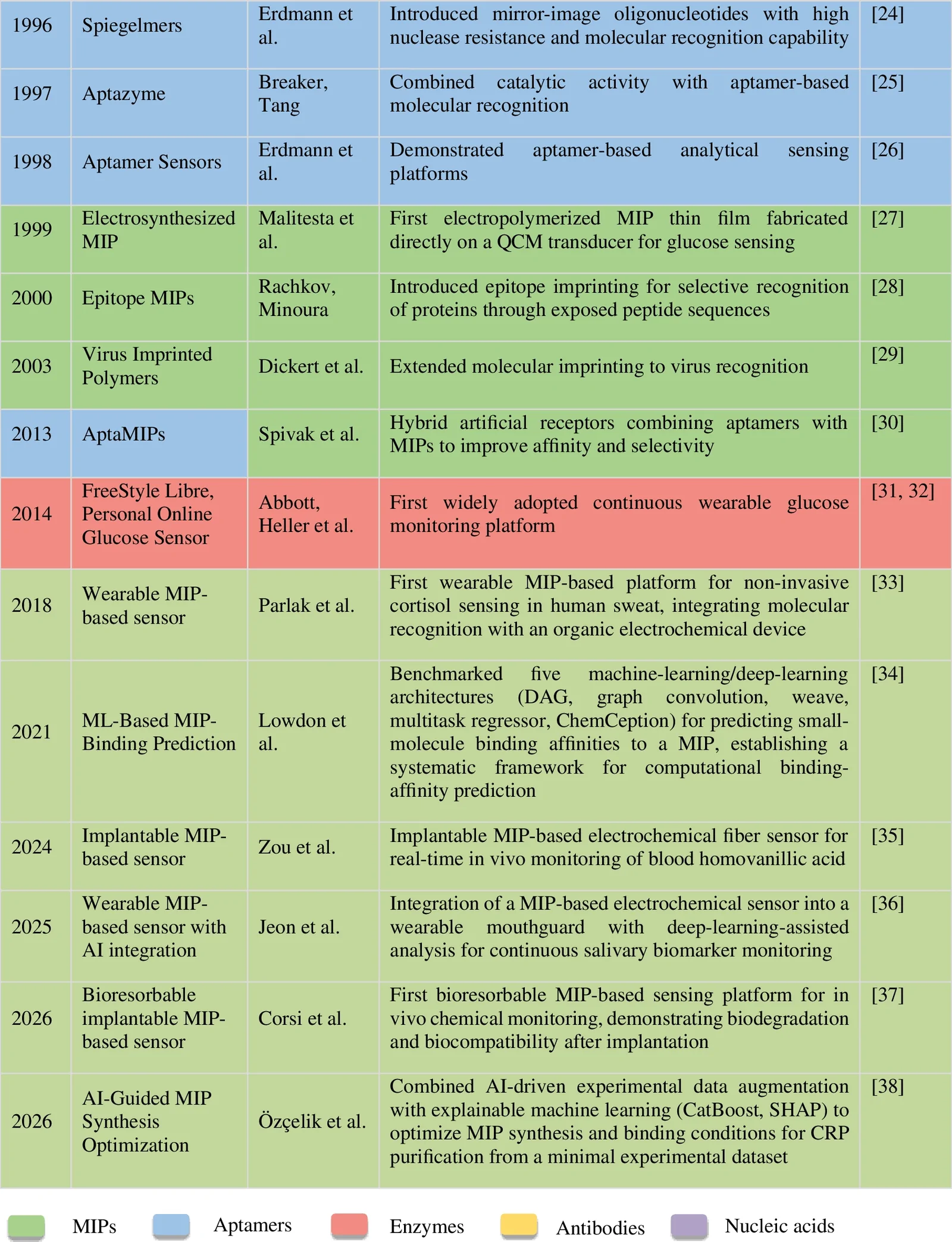
Major milestones in the historical development of bio(mimetic) sensing technologies. Adapted and updated from reference [9]

## Polymers with “molecular memory”

### From bulk imprinting to fully electrochemical MIPs-sensors

**Fig. 1:**
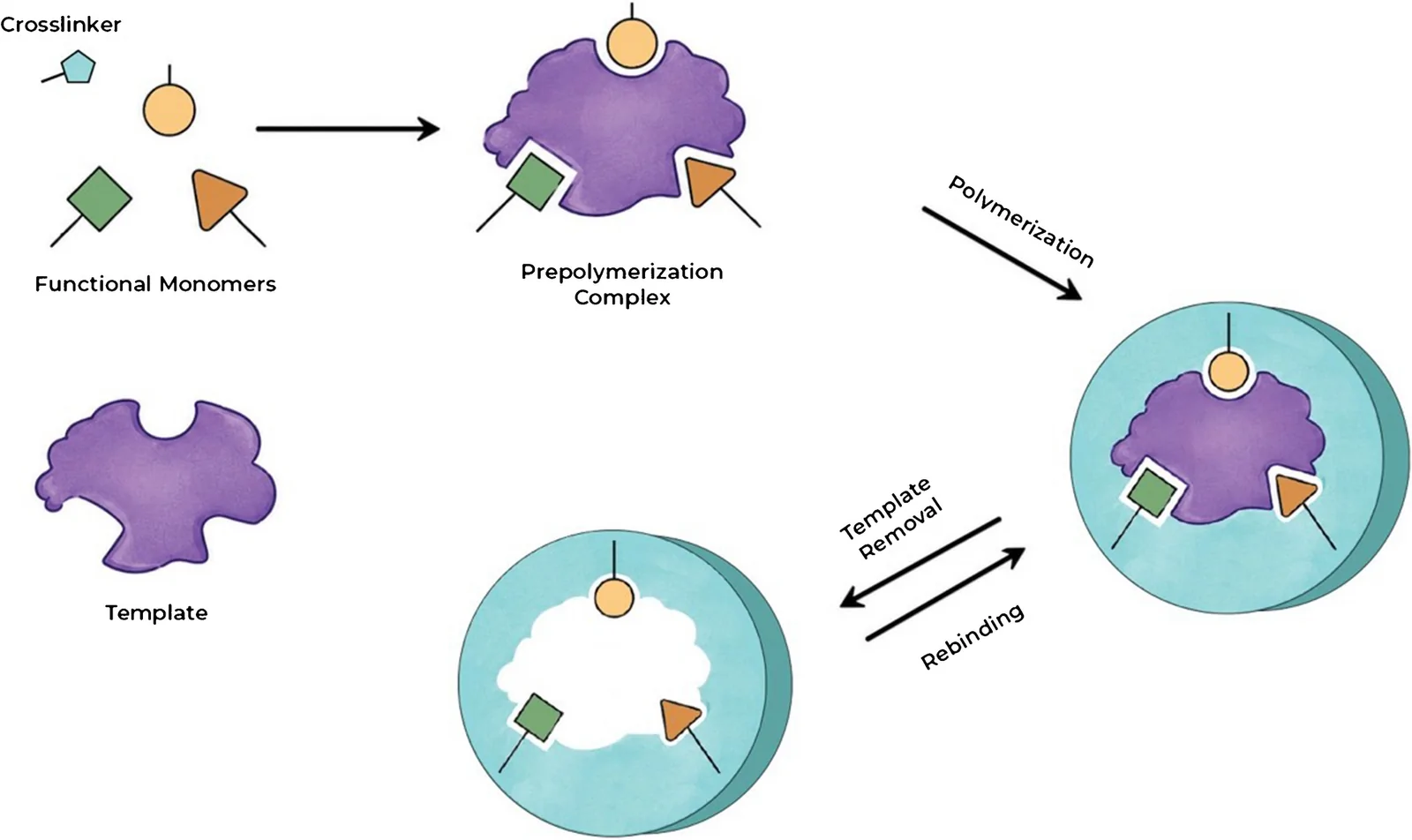
Schematic illustration of the generation of molecularly imprinted polymers (MIPs)

The generation of materials with a molecular memory is an old dream. In this respect, “homeopathy” claims a “**memory**” for biologically active substances in carrier materials (which are “potentiated” by dilution) As early as 1931, the Russian scientist Polyakov produced the first synthetic polymer endowed with a “**molecular memory**” [10]. Prior to polymerization, functional groups of the monomers interact with complementary functionalities of the template, and the template is incorporated into the polymer network during polymer formation (Fig. 1).

**Fig. 2:**
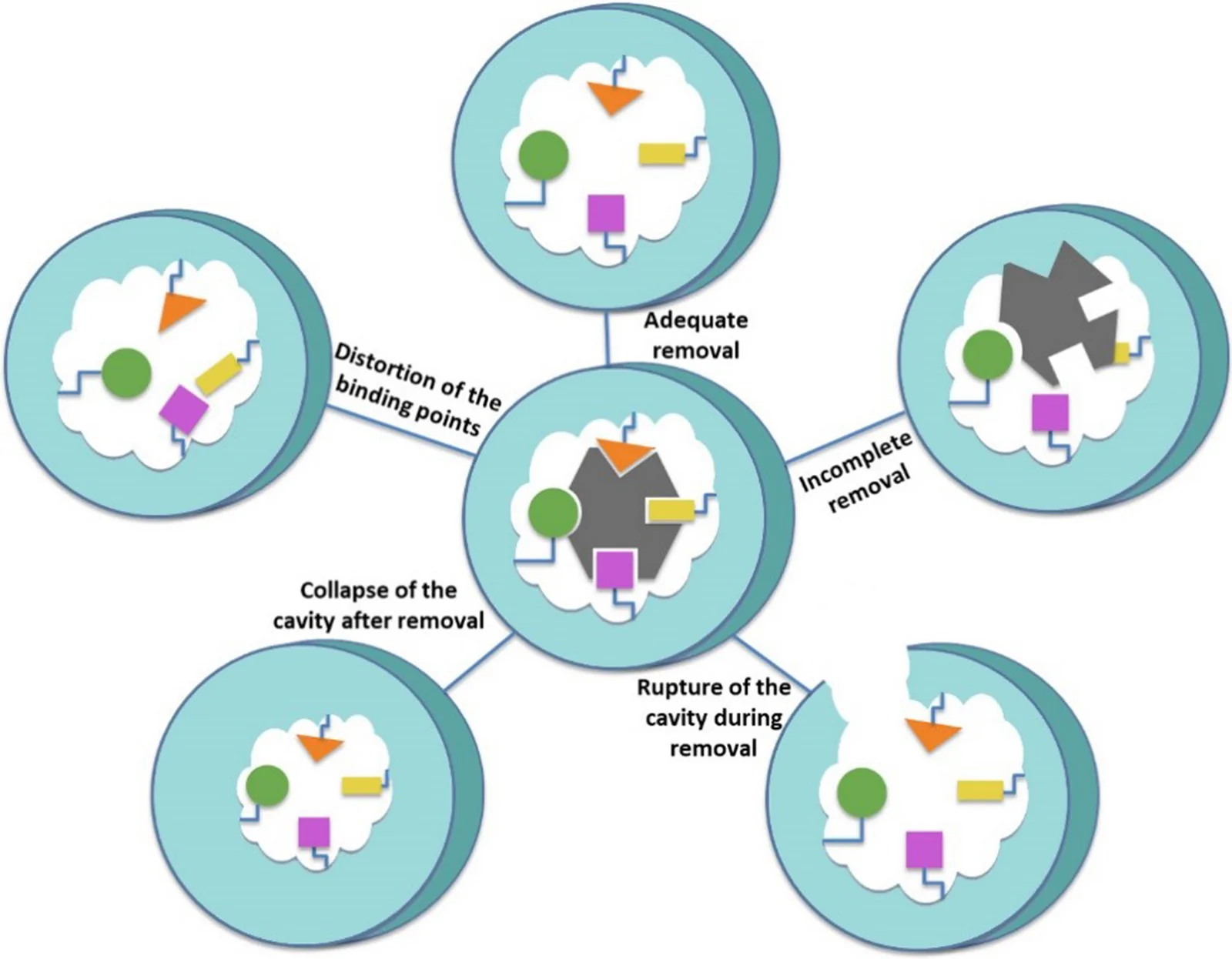
Methods of template removal. Redrawn from reference [39]

Following synthesis, the template must be removed in a gentle yet efficient manner to avoid altering the polymer structure and thereby preserve the template-complementary geometry of the binding sites. Typically, a combination of methods is employed, including temperature elevation, changes in pH or ionic strength, treatment with detergents or organic solvents, as well as electrochemical approaches, which provide a controlled and gentle means of template removal. This process generates cavities with a shape complementary to the target molecule (Fig. 2). These cavities are analogous to the paratopes of antibodies and enable the preferential rebinding of the template.

Only four decades after Polyakov’s concept, Wulff and Mosbach achieved decisive breakthroughs and introduced the term molecularly imprinted polymer (MIP) [2, 3, 40]. Whereas antibodies are composed of the twenty amino acids of proteins, MIP synthesis typically requires only one to five monomers and, where appropriate, the use of a cross-linker. Interestingly, despite their reduced structural complexity, MIPs have demonstrated binding affinities comparable to antibodies in many cases [41, 42]. This observation suggests that high-affinity recognition does not necessarily require the full complexity of biological systems but can instead arise from the precise spatial arrangement of a limited number of functional groups.

**Fig. 3:**
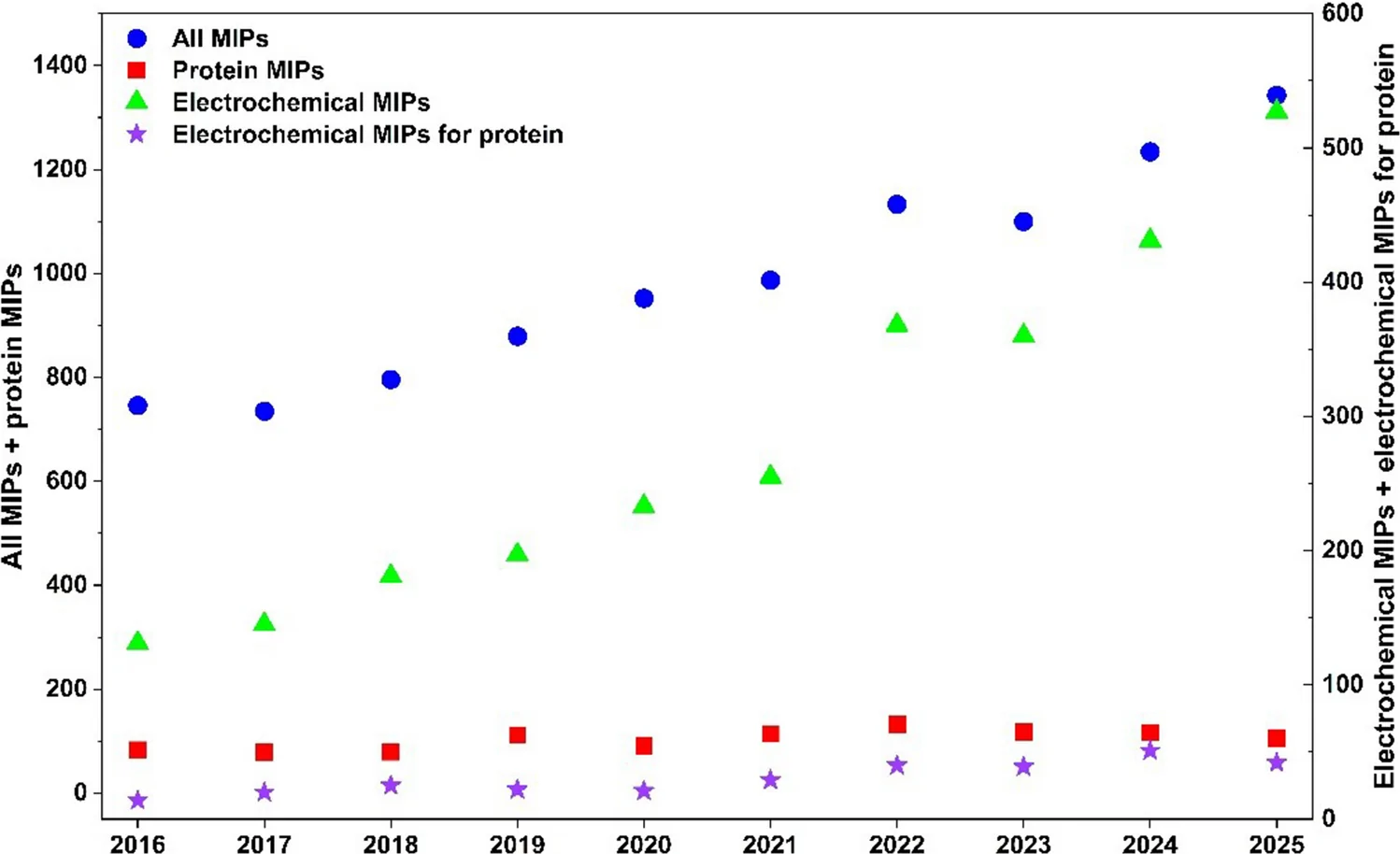
Annual number of publications on MIPs, protein MIPs, electrochemical MIPs, and electrochemical MIPs for protein recognition between 2016 and 2025. Data were obtained from the Scopus database. Blue circles, red squares, green triangles, and purple stars represent the annual publication counts for all MIPs, protein MIPs, electrochemical MIPs, and electrochemical MIPs for protein recognition, respectively

Initially, MIPs could be developed only for small molecules. Today, imprinted polymers are available for the recognition of ions; small molecules such as metabolites, pharmaceuticals and environmental toxins; biomacromolecules; and even whole cells, viruses, and synthetic particles including microplastics [38, 43–53]. As illustrated in Fig. 3, the number of publications related to MIPs has increased substantially over the last decade, rising from 746 publications in 2016 to 1343 in 2025. Although publications on protein MIPs also increased in absolute numbers (from 83 to 106 publications), their proportion of the overall MIP literature remained relatively low (approximately 8–13%) and even declined slightly in recent years, reflecting the ongoing challenges associated with protein imprinting. In contrast, electrochemical MIPs exhibited a much steeper growth, increasing from 131 to 527 publications and expanding their share of the MIP literature from 17.6% in 2016 to 39.2% in 2025. Similarly, electrochemical MIPs for protein recognition increased from 14 to 42 publications during the same period, accounting for approximately 17–44% of all protein MIP publications, highlighting the rapidly growing interest in combining electrochemical transduction with protein imprinting.

Various polymerization techniques have been established for the preparation of MIPs, with bulk imprinting representing the most extensively employed approach. In this method, functional monomers and cross-linkers are co-polymerized in the presence of the template, yielding a rigid, monolithic polymer network. The resulting material is subsequently ground and sieved to obtain particles of appropriate dimensions for the intended application. Despite its operational simplicity and broad applicability, this method presents notable drawbacks, including labor-intensive post-processing steps and the production of irregularly shaped particles that may exhibit slow binding kinetics.

To address these shortcomings, a variety of alternative polymerization strategies have been explored, among them precipitation polymerization, suspension polymerization, emulsion polymerization, and surface imprinting techniques. Surface imprinting strategies such as microcontact printing or electropolymerization are particularly advantageous for macromolecular targets because they generate binding sites at or near the polymer surface, thereby improving accessibility and mass transport properties [54].

In the context of sensor fabrication, two general strategies are typically followed for integrating MIPs with transducers. In the first strategy, the polymer is synthesized separately and subsequently deposited onto the surface of the transducer using techniques such as drop-casting, spin-coating, grafting, or layer-by-layer assembly. In the second strategy, the MIP layer is synthesized directly on the transducer surface (in situ), which allows precise control over film thickness and polymer morphology. Among these methods, electropolymerization has attracted considerable attention because the polymer growth can be finely tuned by controlling the applied potential or total charge during the deposition process [55]. Electrically conducting polymers based on pyrrole, 3,4-ethylenedioxythiophene, ProDOT-COOH, and 2,2′-bithiophene-5-carboxylic acid can be grown into thicker films, which is beneficial for preparing complex three-dimensional micro- or nanostructured MIPs. In contrast, insulating polymer films obtained by the electropolymerization of phenol, o-aminophenol, o-phenylenediamine, and scopoletin generally form polymer layers in the lower nanometer range. However, MIP-synthesis by electropolymerization is limited to a relatively small number of electroactive functional monomers, which may restrict the optimization of template-monomer interactions [56–58].

**Fig. 4:**
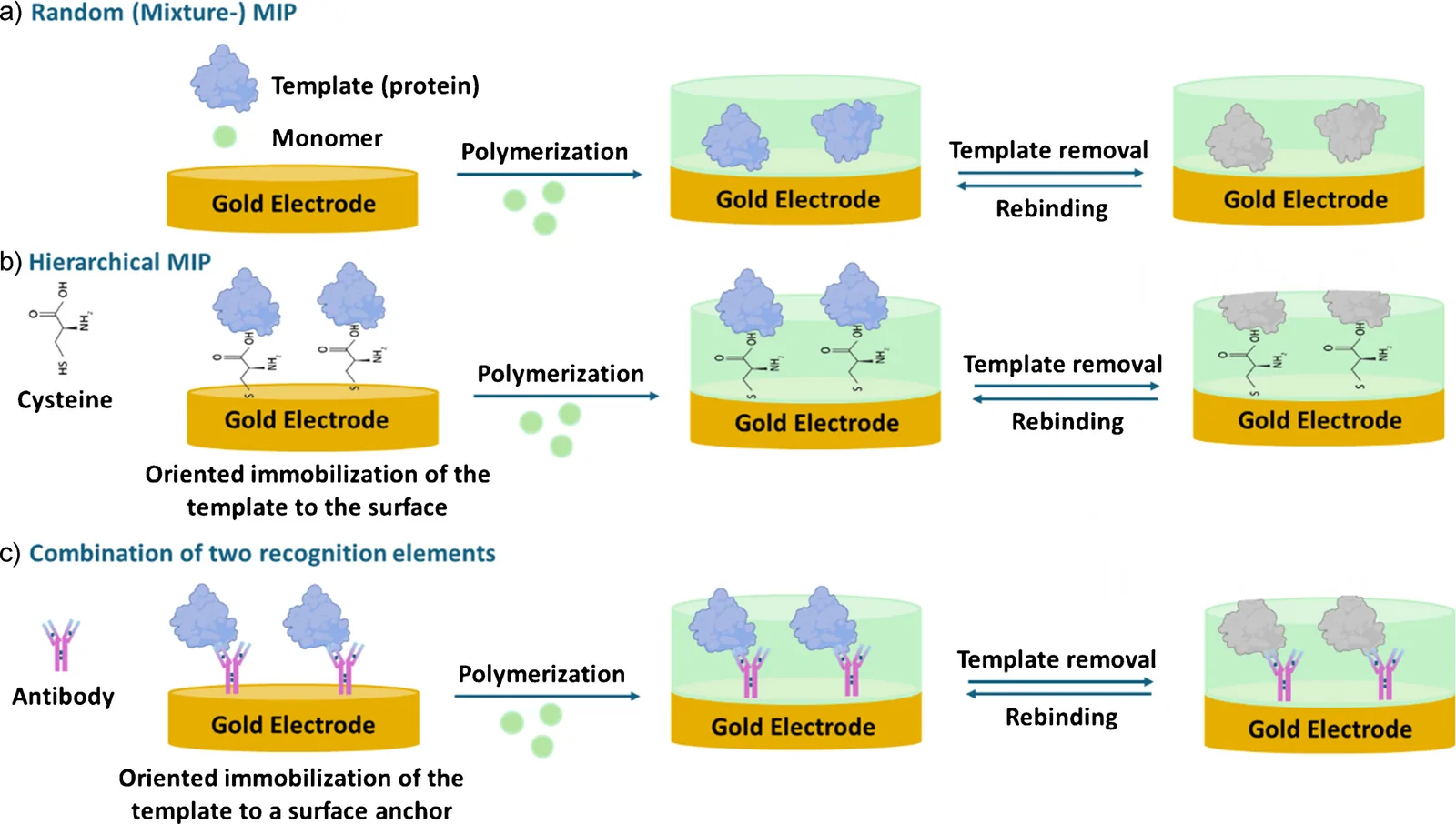
Schematic illustration of different electropolymerization strategies for the fabrication of electrochemical MIP sensors on electrode surfaces: **a** polymerization of a mixture of template and functional monomers to yield random MIPs; **b** generation of hierarchical MIPs by polymerization around an oriented template; and **c** “super-MIPs,” formed by polymerization around a template bound to a surface anchor (antibody, aptamer or lectin) [59]

Within the context of electrochemical MIP sensor fabrication, the arrangement of the template during surface-confined electropolymerization determines the resulting imprinting architecture. Figure 4 illustrates the principal electropolymerization strategies for fabricating electrochemical MIP sensors on electrode transducers [59]. Polymerization of a mixture of monomers and template represents the simplest route to MIP synthesis (Fig. 4a). In this approach, the template molecules are randomly distributed within the pre-polymerization mixture, resulting in binding cavities with random orientation, geometry, and accessibility (random MIPs). The resulting heterogeneity of binding sites complicates template removal and creates quality issues in large-scale production. In contrast, hierarchical MIPs are prepared by first immobilizing and orienting the template molecules on the transducer surface before polymerization, producing more homogeneous and uniformly oriented binding cavities (Fig. 4b) [60]. Polymerization around oriented, immobilized low molecular weight species—and in particular macromolecules—results in higher affinities compared with random MIPs. To combine the advantages of two recognition elements, MIPs have been spatially combined with antibodies, lectins, or aptamers to form so-called super-MIPs (Fig. 4c). In this approach, MIP synthesis is performed around surface-bound complexes of template and binder. To date, this concept has yielded only modest improvements in affinity and is considerably more complex in preparation than conventional MIPs.

These different synthetic approaches provide significant flexibility for tailoring MIP properties according to the intended analytical or catalytic application. Consequently, MIPs have been successfully developed for a wide variety of targets, from low molecular weight compounds to large molecular structures. Despite these advances, challenges remain in understanding how polymer cavities can accurately reproduce the structural features of templates that are significantly smaller than the polymer building blocks, particularly in the case of ions or small molecules [61].

### Imprinting of substructures: epitopes, tags, and labels

Inspired by the mechanism of antigen-antibody recognition of macromolecules, Rachkov and Minoura introduced the “epitope concept” for MIPs: similar to antibodies, MIPs recognize only a substructure (i.e., a fragment) located on the surface of the target molecule, while still enabling the binding of the entire macromolecule [62]. The principle of epitope imprinting was first demonstrated for the nonapeptide oxytocin, in which the C-terminal tetrapeptide was used as the template, and the resulting MIP, after template removal, was able to bind the full peptide hormone with comparable affinity. Modelling studies further demonstrated that peptides consisting of as few as three amino acids can already produce affinities in the micromolar range [63]. However, for achieving high selectivity, longer peptide sequences are generally required; therefore, templates consisting of six to fifteen amino acids are typically preferred [64].

Most commonly, N- or C-terminal peptides have been used to generate MIPs for protein recognition, for example in the case of cytochrome c and bovine serum albumin. Following this approach, we compared the performance of random and hierarchical MIPs using the C-terminally cysteine-extended StrepTag II peptide (WSHPQFEKC) and the N-terminal peptide of the β-chain of hemoglobin (CVHLTP) as templates [65, 66]. For the target peptide, the *K*_d_ value of the hierarchical StrepTag MIP (2.54 nM) was approximately ten-fold lower than that of the random MIP, while for the VHLTP MIP, it was more than 20-fold lower than that of mixture-based MIPs (64.6 nM vs. 1.4 µM). Moreover, both hierarchical MIPs exhibited higher affinities toward their parent proteins. Evidently, the predominantly vertical orientation of the binding cavities is advantageous for the recognition of both the template peptide and the terminal epitopes present in the parent proteins, namely Strep-Tag II hydrogenase and hemoglobin. These findings demonstrate the superiority of the hierarchical MIP concept, despite its more elaborate fabrication. Nevertheless, this approach remains challenging because the structure of the “free” peptide used as the template is inherently different from that of the epitope on the surface of the parent protein molecule.

MIPs have also employed the C-terminally cysteine-extended nonapeptide SKTRAALGVC (SK peptide) in an electrochemical sensor for the 69 kDa glycoprotein α-fetoprotein (AFP), as well as the G peptide GFNCYFP as a template for the detection of the receptor-binding domain (RBD) of SARS-CoV-2 [67]. The *K*_d_ values of the epitope MIPs for AFP (13.2 nM) and RBD (14.7 nM) were comparable to those of MIPs prepared using the entire protein as the template (14.5 nM for AFP and 16.8 nM for RBD, respectively). Moreover, the epitope MIPs exhibited lower non-specific binding toward human serum albumin. Evidently, the smaller binding sites of epitope MIPs confer higher selectivity than protein-imprinted polymers. The comparable affinities, reduced non-specific binding, and lower template costs constitute major advantages of epitope MIPs over protein-imprinted counterparts.

Following the paradigm of antibody binding to non-terminal epitopes, MIPs have also been successfully generated using exposed internal sequences. From a structural perspective, however, it is difficult to understand how a “flat” epitope on the protein surface can effectively bind to the “perpendicularly” oriented cavities formed by cysteine-terminated templates [68]. To create more “horizontal” binding pockets for protein recognition, Altintas et al. [69] used a 12-amino-acid-long epitope derived from neuron-specific enolase (NSE) of the human brain, extended with cysteine residues at both termini, as the template. The doubly cysteine-modified peptide templates formed self-assembled monolayer epitope linkers on gold surfaces, enabling subsequent molecular imprinting through electropolymerization for the detection of short peptides or the NSE protein. According to the proposed scheme, effective recognition of the peptide appears plausible; however, it remains difficult to rationalize how NSE itself can bind to the MIP with a K_d_ value below 65 pM.

Haupt and co-workers designed a cyclic template based on the highly conserved 3S epitope of the HIV-1 gp41 protein by introducing two terminal cysteine residues to form a disulfide bridge and an additional glycine residue to stabilize the cyclic conformation [70]. The cyclic peptide was immobilized via its terminal carboxyl group on amino-functionalized glass beads in an oriented configuration and subsequently used for the solid-phase synthesis of nanoMIPs. The resulting nanoparticles (65 ± 4 nm) exhibited a dissociation constant of 79.6 nM toward the cyclic peptide and retained high affinity for the corresponding gp41 protein (IC₅₀ = 85.4 nM). Moreover, the cyclic template showed approximately three-fold higher affinity than the corresponding linear peptide (IC₅₀ = 18.7 vs. 69.0 nM), demonstrating that conformationally constrained peptide templates constitute an effective alternative for improving epitope recognition.

**Fig. 5:**
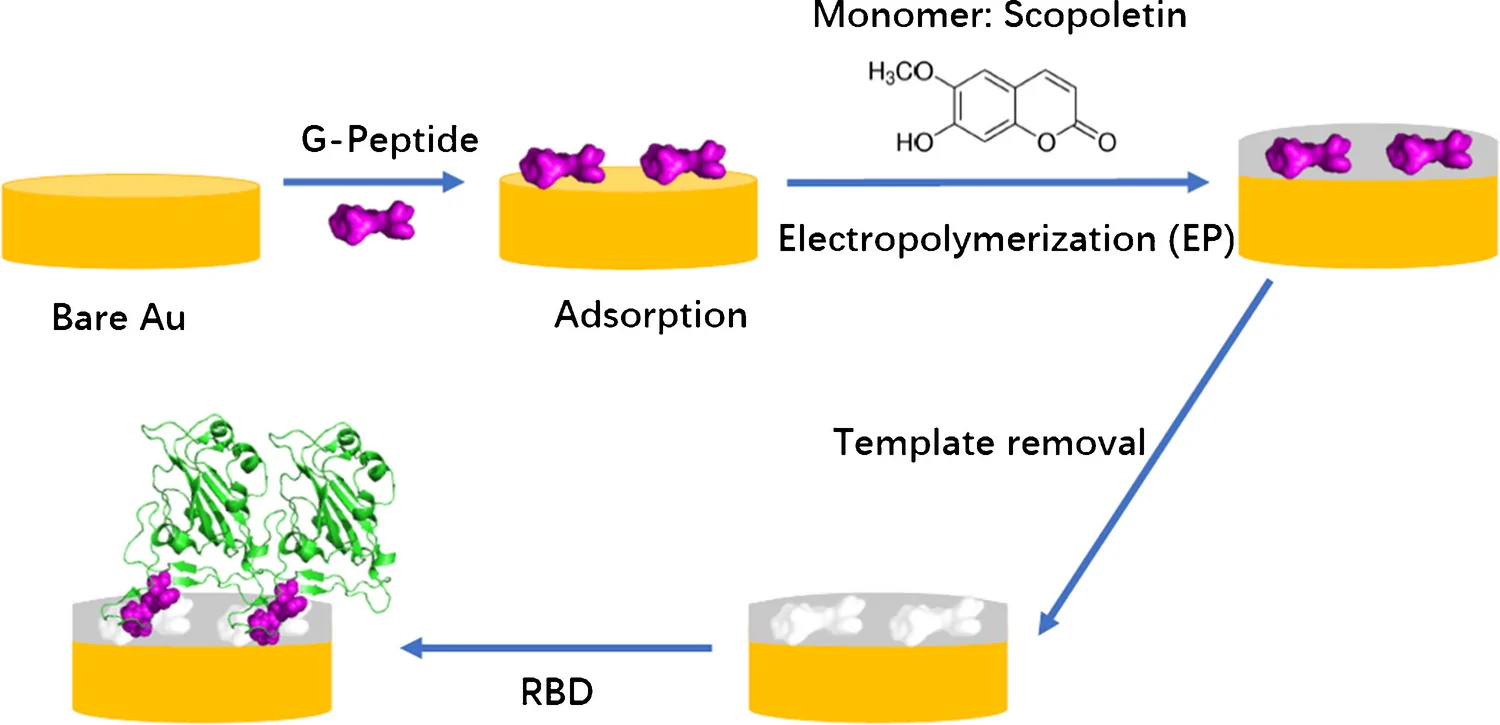
Fabrication of an epitope-imprinted polyscopoletin nanofilm using a horizontally oriented G-peptide template on a gold electrode and subsequent recognition of the SARS-CoV-2 receptor-binding domain (RBD). Reproduced from ref. [71]

For effective recognition of the parent protein by “flat” cavities, we applied a peptide containing a central cysteine, which was expected to chemisorb onto the underlying gold electrode in a horizontal orientation [71]. The heptapeptide 485–491 GFNCYFP (containing the central Cys488) from the binding site of the SARS-CoV-2 RBD to the ACE2 receptor of the host cell was used as the template. The selectivity of the G-peptide-imprinted polyscopoletin nanofilms was investigated through binding studies using different peptides containing amino acid motifs derived from the G-peptide template. The highest affinity was observed for the template epitope G-peptide (*K*_d_ = 58 nM). The contribution of the central cysteine (Cys488 in the native protein) to molecular recognition was evaluated for rebinding to the G-peptide MIP using an analogous peptide in which Cys488 was replaced by serine. This single amino acid substitution resulted in an approximately 15-fold decrease in affinity, corresponding to a 15-fold higher *K*_d_ value of 859.6 ± 0.1 nM. Finally, the N-terminal tetrapeptide EGFN showed no binding to the G-peptide-imprinted polymer. These results are consistent with a previous finding by Piletsky’s group that at least four common amino acids with the target are required for effective peptide binding to the MIP. The fabrication and recognition principle of this epitope-imprinted nanofilm is summarized in Fig. 5.

The epitope concept has been successfully transferred to recombinant proteins carrying affinity tags (e.g., His₆, FLAG/DYKD, and Strep tags), where the tag itself serves as the template [65, 72, 73]. A substantial extension of this approach is provided by imprinting substructures such as dye labels of nucleic acids, surface proteins or carbohydrate moieties of viruses and cells. This strategy exploits the advantages of imprinting small molecules—greater stability, lower cost relative to biomacromolecules or bioparticles, and easier template removal. By employing several low molecular weight substructure templates, “multi-analyte” sensors can be realized. Representative examples of epitope-imprinted protein MIPs, including template design, selectivity profiles, analytical ranges, and binding affinities, are summarized in Table S1.

### Anti-idiotypic MIPs

**Fig. 6:**
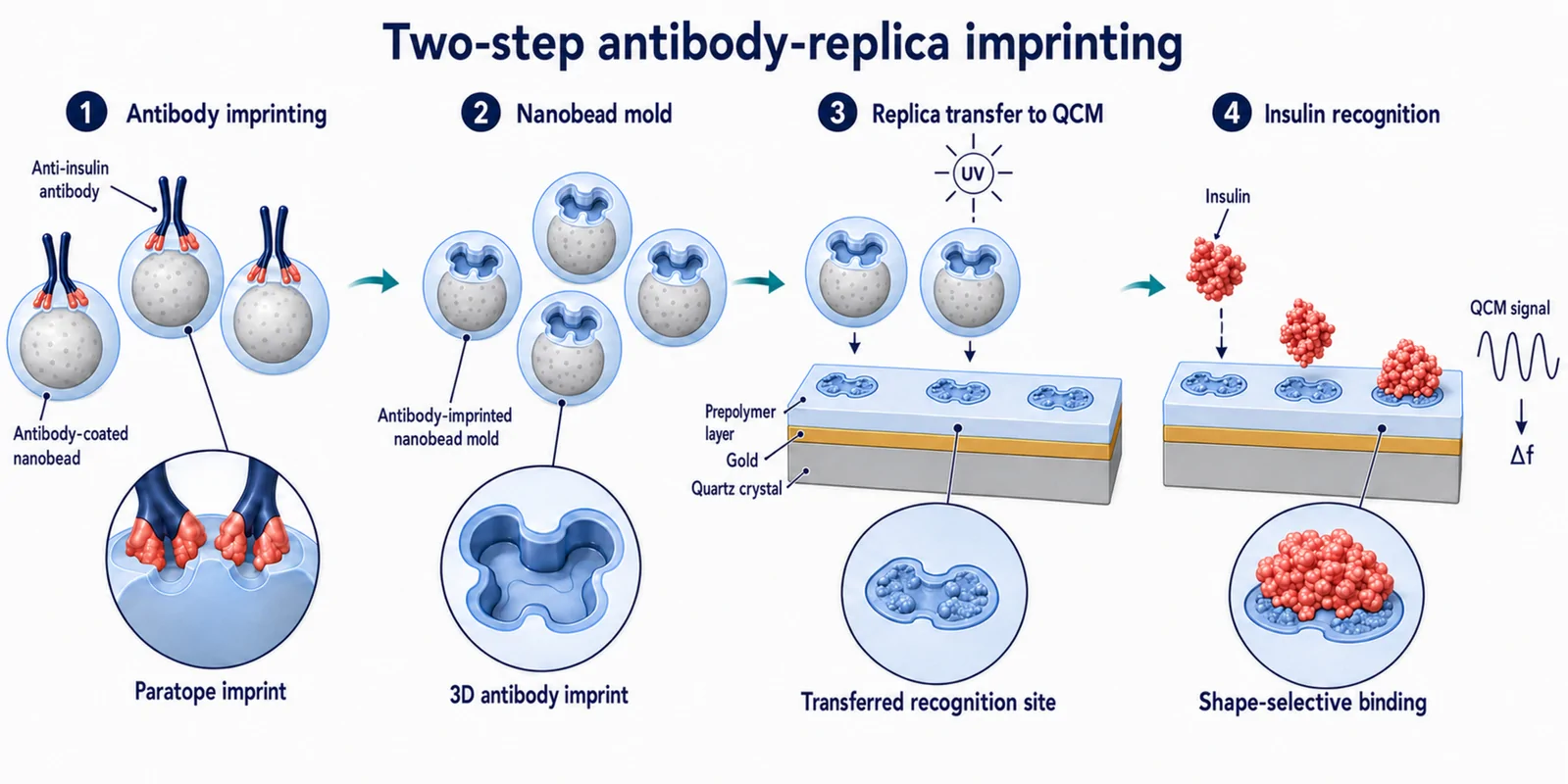
Schematic representation of the antibody-replica approach used to generate anti-idiotypic MIPs for insulin sensing. Adapted from Ref. [74]. Generative AI was used to assist in the creation of this schematic illustration

Dickert’s group transferred the concept of anti-idiotypic antibodies to the synthesis of anti-idiotypic MIPs [74]. Following Mosbach’s idea of “double imprinting,” they synthesized a “replica” of an anti-insulin antibody. In the first step, antibody molecules were imprinted onto the surface of nanoparticles. After removal of the template, the nanoparticles carried cavities resembling the shape of the antibodies, including the antigen-binding region (the paratope). In the second imprinting step, these particles were pressed into a thin layer of pre-polymerization gel deposited on the sensor surface. After polymerization and removal of the antibody-imprinted particles, cavities replicating the shape of the original antibody, including the paratope, were formed. Interaction with 10 µM insulin resulted in a signal that was 1.5-fold higher than that of an insulin-imprinted polymer and a 30-fold stronger QCM signal than that of the non-imprinted polymer. At saturation concentration, a monolayer of tetrameric insulin was bound. Cross-reactivity studies with glargine, which differs from insulin by only two amino acids, showed nearly half the affinity compared with insulin binding. The two-step antibody-replica concept is illustrated in Fig. 6.

### Post imprinting modification (PIM)

**Fig. 7:**
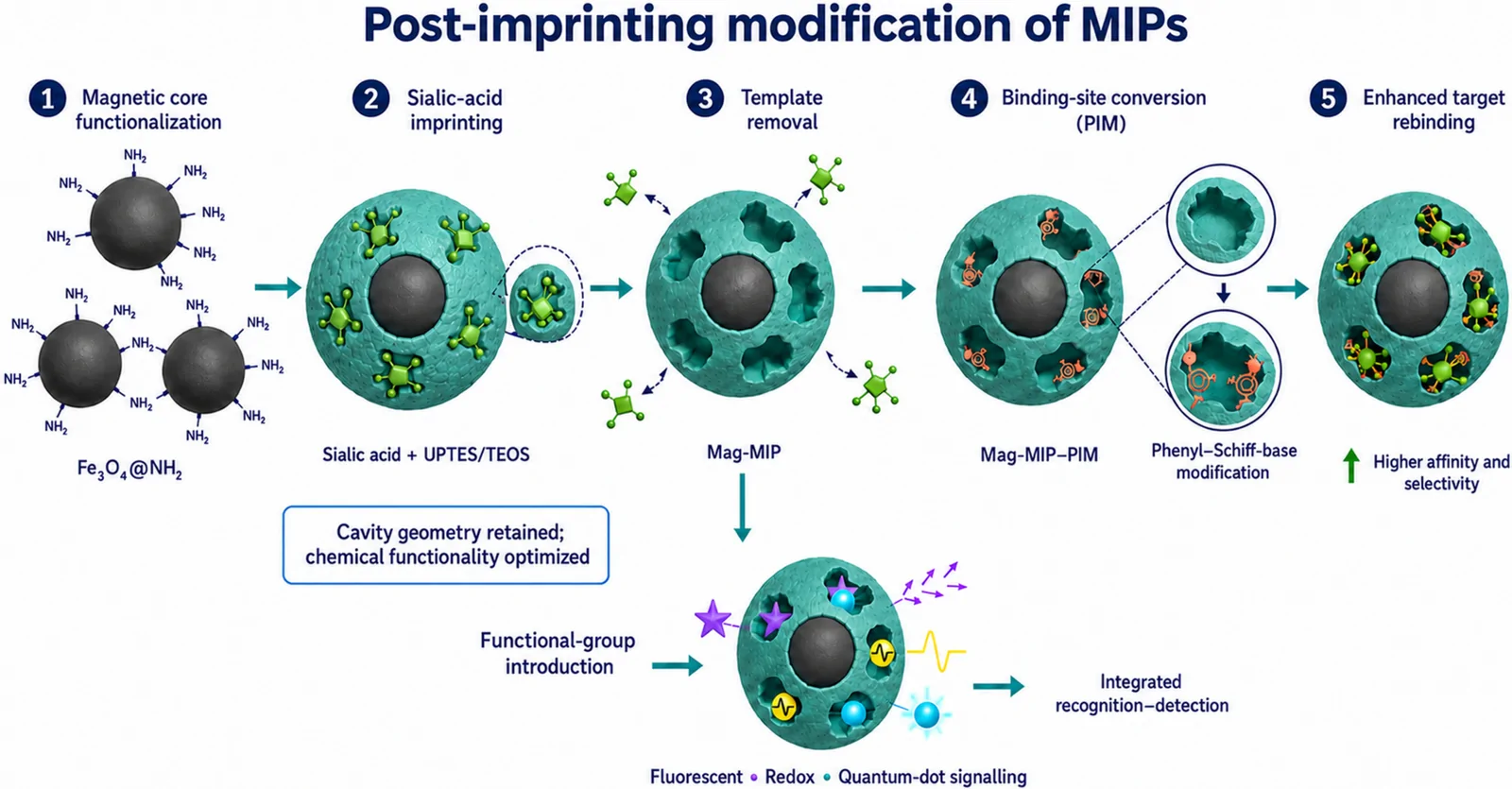
Preparation of magnetic MIPs and phenyl-Schiff-base post-imprinting modification for the selective recognition of sialic acid and sialylated glycoproteins. Adapted from Ref. [77]. Generative AI was used to assist in the creation of this schematic illustration

Post imprinting modification (PIM) is inspired by protein post-translational modifications in cells. It typically involves introducing specific chemical functionalities after template removal into pre-formed MIP cavities [75, 76]. By directionally altering or introducing new functional groups, the recognition properties of MIPs can be improved while the predetermined cavity structures are retained. PIM approaches can be classified into binding site conversion and functional group introduction. Using binding site conversion functional groups within the imprinted cavities are modified in order to introduce high-affinity binding toward target molecules. An example is the incorporation of phenyl-Schiff base groups into the cavities of a sialic acid–imprinted polymer which resulted in enhanced affinity and selectivity for sialic acid and sialylated glycoproteins [77]. The functional group introduction incorporates novel “signalling” units such as fluorescent molecules, redox markers or quantum dots to integrated “recognition-detection” functionality [78, 79]. A representative post-imprinting modification strategy for sialic acid recognition is shown in Fig. 7.

## Integration of nanomaterials to MIP sensors

In recent years, the role of nanomaterials in MIP-based sensors has expanded significantly. By increasing the active surface, improving mass transport, charge transfer, surface accessibility, and structural control, these materials have enabled the development of more efficient sensing platforms [80, 81]. In addition, plasmonic and photocatalytic nanoparticles allow direct signal generation upon target binding to the MIP. Representative examples of nanomaterial-assisted MIP sensing platforms, including transduction methods, target analytes, and principal analytical advantages, are summarized in Table S2.

### Nanoparticles

Nanoparticles (NPs), particularly metallic and metal oxide nanoparticles, play a central role in surface imprinting approaches. Their use in MIPs is primarily motivated by their ability to provide high surface area, tunable surface chemistry, and enhanced electron transfer for signal generation [82]. Gold (Au) [83], silver (Ag) [84], platinum (Pt) [85], iron oxide (Fe₃O₄) [86], titanium dioxide (TiO₂) [87], and zinc oxide (ZnO) [88] nanoparticles are widely used as core materials for the formation of thin imprinted shells. Au NPs are particularly advantageous due to their ease of functionalization (e.g., via thiol chemistry), enabling oriented immobilization of templates such as peptides and proteins, which leads to more homogeneous binding sites [89, 90]. Ag NPs provide plasmonic enhancement (SERS) for signal amplification [84], while Pt NPs act as electrocatalysts that accelerate redox reactions and increase current response [80]. Metal oxide nanoparticles such as TiO₂ and ZnO exhibit semiconducting properties and photocatalytic activity, while providing stable and adsorption-active surfaces, thereby supporting both binding site formation and signal transduction [88, 91]. Fe₃O₄ nanoparticles are distinguished by their magnetic properties, which have led to their widespread use in magnetic solid-phase extraction (MSPE) [92] and magnetically separable MIP systems [93]. Furthermore, their intrinsic peroxidase-like activity has promoted the development of MIP-based nanozyme sensing platforms [94].

Nevertheless, the analytical performance of NP-assisted MIPs depends strongly on the rational design of the hybrid architecture rather than on the incorporation of nanoparticles alone. Parameters including nanoparticle size, morphology, loading, surface functionalization, the combination strategy, and the thickness of the imprinted shell directly influence particle dispersion, accessibility of recognition sites, and mass-transfer kinetics, thereby affecting both sensitivity and selectivity. Furthermore, non-uniform nanoparticle distribution, poor interfacial compatibility between the nanoparticle core and the MIP layer, and non-specific adsorption may compromise reproducibility and analytical performance, particularly when complex sample matrices are analyzed. Therefore, metallic/metal oxide nanoparticles should be selected according to the sensing mechanism and fabrication strategy, as their contribution to sensor performance is highly application-dependent and requires careful optimization of the NP-MIP interface [95, 96].

### Carbon-based nanomaterials

Carbon-based nanomaterials have become widely used in MIP-based electrochemical sensors because they provide a conductive and easily modifiable interface between the imprinted polymer layer and the electrode [97]. In recent studies, reduced graphene oxide [98, 99], carbon nanotubes [100, 101], laser-induced graphene [102], and graphene-based composites [103, 104] have been increasingly employed to increase electroactive surface area, improve interfacial stability, and support the development of miniaturized sensing platforms. The recent work of Xu et al. illustrates this point particularly well. They developed a laser-induced graphene (LIG)–based electrochemical sensor incorporating an electropolymerized MIP layer for the ultra-trace detection of perfluorooctanoic acid (PFOA). The sensor exhibited a limit of detection of 0.025 ppt over a linear range of 0.025 ppt to 25 ppb and maintained selectivity in the presence of structurally related PFAS compounds and common ions [102].

The integration of carbon nanomaterials into MIPs requires careful control over both the support material and the imprinting process. For CNT-based MIPs, surface oxidation or chemical functionalization is generally required to introduce carboxyl groups for polymer grafting; however, harsh oxidative treatments may disrupt the graphitic structure and reduce electrical conductivity. Likewise, although graphene derivatives provide abundant oxygen-containing functional groups, excessive oxidation may compromise electrical transport, whereas insufficient functionalization can result in poor polymer anchoring. Therefore, the analytical performance of carbon-based MIPs is determined not only by the intrinsic properties of the carbon material but also by the balance between surface functionalization, preservation of the carbon framework, and accessibility of the imprinted recognition sites after polymerization [105].

### Metal-organic frameworks (MOFs)

Metal-organic frameworks are especially attractive in this context because they combine crystallinity, high surface area, tunable pore size, and chemically programmable coordination environments [106]. In hybrid MOF/MIP materials, the MOF does not simply serve as an inert carrier; rather, it acts as a porous scaffold that increases the accessible surface for imprinting, shortens diffusion pathways, and can participate in signal generation, particularly in electrochemical [107] and fluorescence formats [108]. Recent reviews have emphasized that the synergy between the selective recognition of MIPs and the porosity and functional tunability of MOFs improves mass-transfer efficiency, adsorption selectivity, and signal amplification, especially for analytes that are difficult to detect in complex matrices [109–111]. In addition, MOF-supported or MOF-derived imprinted layers can expose a larger fraction of binding sites to the sample phase, further improving target accessibility and rebinding efficiency [112, 113]. Recent literature shows that such systems are now being applied far beyond proof-of-concept studies, including food contaminants [109], antibiotics [114], mycotoxins [115], and microbial targets [110].

An important consideration in the design of MOF/MIP composites is the compatibility between MOF synthesis and the molecular imprinting process, since both MOF activation and template removal can affect the structural integrity of the hybrid architecture. Furthermore, the compatibility of MOF/MIP composites with a particular transducer or transduction mechanism should be assessed for each sensing platform. Consequently, these hybrid architectures are particularly valuable in sensing platforms requiring analyte preconcentration, secondary transduction, or charge-transfer processes [116].

### MXenes

More recently, MXenes, a family of two-dimensional transition metal carbides and carbonitrides, have emerged as an additional class of conductive materials in MIP-based sensors. Unlike graphene-based materials, MXenes possess surface terminal groups such as –OH, –O, and –F, which improve hydrophilicity [117]. Among them, Ti₃C₂Tₓ is one of the most frequently investigated MXenes in electrochemical MIP platforms because of its high electrical conductivity, layered morphology, and large accessible surface area [117, 118]. Recent studies have applied Ti₃C₂Tₓ-based MIP sensors to pharmaceutical analysis, environmental monitoring, and biomarker detection [119–121].

Despite the promising performance of MXene-based MIPs, their broader implementation is limited by the reliance on only a few MXene compositions (mainly Ti₃C₂Tₓ and Ti₂C) and the need for greener synthesis routes that avoid hazardous etchants such as HF. Moreover, simplified fabrication strategies and validation under practical sensing conditions remain necessary for their translation toward point-of-care and commercial applications [122].

## MIP sensor arrays

The inherent tunability of molecularly imprinted polymers makes them particularly well-suited for integration into sensor array platforms, which also enables simultaneous multi-analyte detection. This approach mirrors the conceptual architecture of immunosensor arrays and DNA microchips but replaces biological recognition elements with synthetic, thermally and chemically stable polymer cavities.

Readout of MIP sensors and arrays employs the same methods and assay formats as immunosensors or DNA chips, including fluorescence, SPR, and QCM, but in particular a variety of voltammetric techniques.

As early as 2005, Greene and Shimizu demonstrated a seven-MIP colorimetric sensor array using a dye-displacement readout that accurately classified seven aromatic amines with 94% accuracy, illustrating the viability of MIP arrays for pattern-based identification across a set of structurally related compounds [123]. Stephenson and Shimizu further emphasize MIP arrays as a robust approach when a single MIP element exhibits limited selectivity, arguing that an array can deliver high discrimination through collectively distinct binding patterns [124].

Lin et al. [125] reported a four-channel photonic crystal molecularly imprinted polymer (PCMIP) sensor array for the simultaneous discrimination of multiple sulfonamide antibiotics (SAs) The array comprised four sensing units: three prepared with sulfaguanidine (SG), sulfamethazine (SM2), and sulfathiazole (ST) as respective template molecules (designated SG-PCMIP, SM2-PCMIP, and ST-PCMIP), and one non-imprinted control unit (PCNIP) fabricated in the absence of any template. The array was evaluated against six SAs—SG, SM2, ST, sulfadiazine (SD), sulfadimethoxine (SDM), and sulfanilamide (SA)—across three concentration levels (10⁻^4^, 10⁻⁶, and 10⁻⁸ mol/L). At 10⁻^4^ mol/L, each imprinted unit produced a pronounced response toward its corresponding template, consistent with the presence of complementary recognition cavities, while the PCNIP unit exhibited the lowest sensitivity and selectivity across all six analytes. Array performance in a real food matrix was assessed by analyzing spiked fish samples. Predicted and measured LDA regions showed strong spatial agreement, yielding a discrimination accuracy of 90.9% despite interference from complex matrix components. These results establish the PCMIP array as a viable strategy for multi-residue sulfonamide screening in food safety applications.

**Fig. 8:**
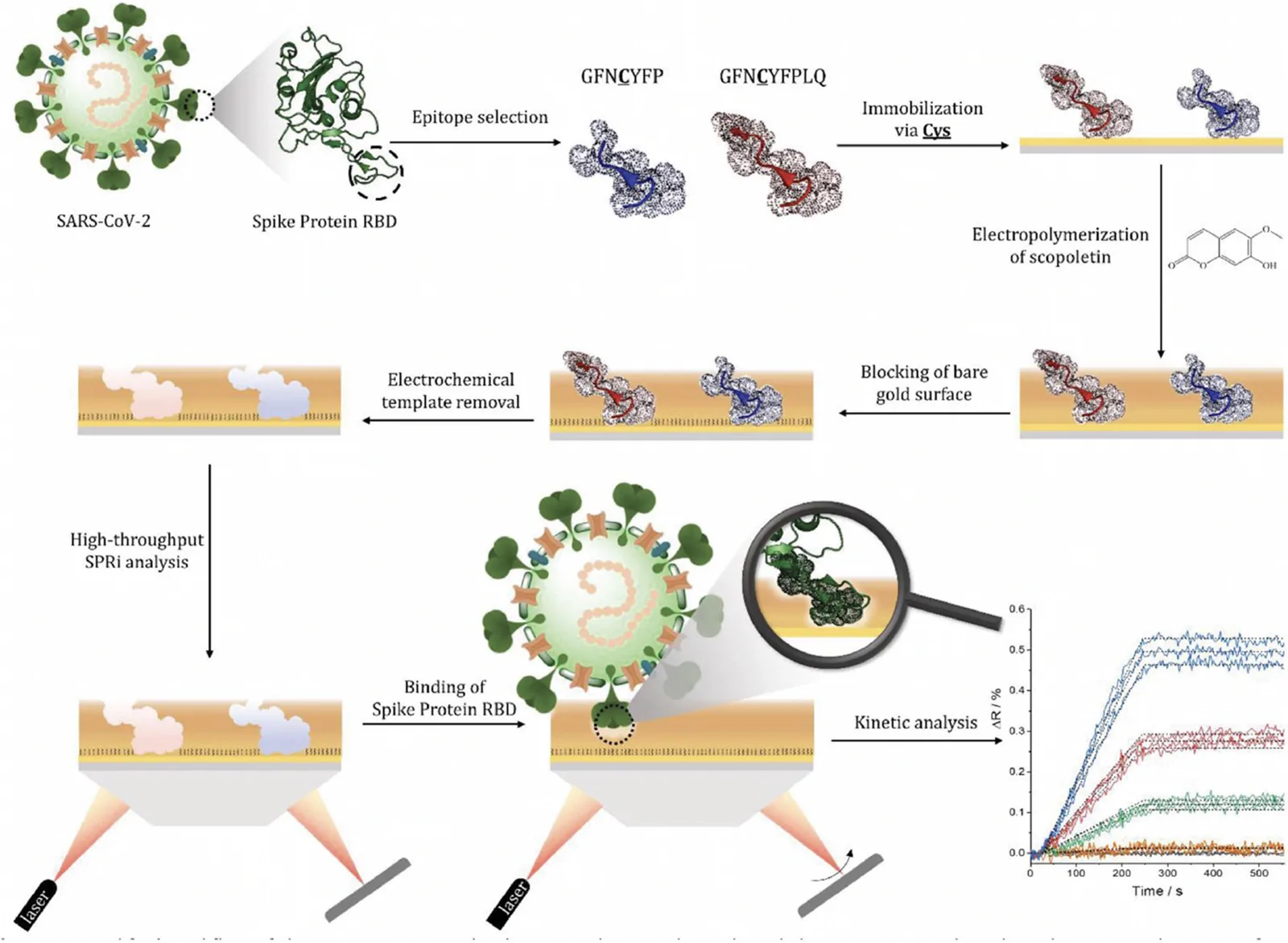
Schematic representation of the fabrication of an MIP array comprising two epitopes of the spike protein and SPR-based readout of viral binding via the RBD [130]

During the global COVID-19 pandemic, immunochromatographic rapid tests became the method of choice for patient self-testing prior to PCR confirmation. In this period, MIP sensors were successfully developed using (inactivated) viruses [126], the spike or nucleocapsid protein [127, 128], the receptor-binding domain (RBD) [129], and epitope peptides [130] as templates. In collaboration with partners at the Budapest University of Technology and Economics (Hungary), we developed fully electrochemically prepared MIP-SPR arrays based on short peptides derived from the viral binding region of the spike protein interacting with the host cell receptor. The peptide epitope GFNCYFP, corresponding to a segment of the receptor-binding domain critical for ACE2 interaction, was selected as the imprinting template. The peptide was chemisorbed onto the gold transducer surface via its central cysteine residue, followed by the formation of a thin polymer matrix around the template by electropolymerization. Template removal was achieved by applying anodic potential pulses, resulting in accessible imprinted cavities with well-defined three-dimensional complementarity to the peptide epitope. This approach is conceptually analogous to microelectrospotting-based strategies, enabling spatially controlled localization of recognition sites on the transducer surface. Notably, this principle was applied for the first time by our joint research team using ferritin as a template, demonstrating the feasibility of extending spatially controlled imprinting strategies to larger biomacromolecular systems. The (re)binding of the peptide, the RBD, the spike protein and virus-like particles was monitored either directly by surface plasmon resonance (SPR) (Fig. 8) or via changes in the permeability of the MIP nanofilm for the redox marker ferricyanide as a function of binding site occupancy [130].

In buffer containing a large excess of human serum albumin, as well as in the “system solution” of commercial COVID-19 antigen rapid tests, RBD was detected in the lower nanomolar concentration range. In parallel, the British company MIP Discovery announced a thermo-resistive COVID sensor in 2021 that likewise employs a peptide from the RBD binding region as a template.

## Catalytically active MIPs

MIPs have been developed not only for analyte binding but also as mimics of enzymes and catalytic antibodies [131–143]. One strategy for generating catalytically active MIPs is the use of a stable analogue of the transition state of the reaction to be catalyzed as the template during MIP synthesis. For example, we developed an MIP that catalyzes the hydrolysis of carbamates and achieved a specific activity of 211.15 ± 20.16 nmol min^−1^ mg^−1^ [144]. Another concept is based on incorporating “nanozymes” into a substrate-imprinted polymer network. To this end, transition metals such as Fe(III), Cu(II), and Zn(II), as well as their complexes, or enzymatic catalytic centers such as heme and selenocysteine, are embedded within the MIP [145–147]. In this configuration, the polymer assumes the role of the apoenzyme, while the artificial enzyme provides the catalytic center. To date, catalytically active MIPs typically achieve less than one percent of the activity of natural enzymes catalyzing the same reaction [135]. Interestingly, catalytic MIPs have been developed for reactions—for example the Diels-Alder reaction—for which no natural enzymes exist [148]. Table S3 summarizes representative examples reported since 2020 on the synergistic integration of enzymes or enzyme-mimicking systems with MIP-based electrochemical sensors. The listed studies highlight three main approaches: regenerable enzyme-MIP hybrids, catalytically active (enzyme-like) MIPs, and enzyme-assisted signal amplification strategies.

Beyond sensing, MIPs have also been realized for applications such as protein stabilization, crystallization, and refolding. Takeuchi et al. demonstrated that an MIP templated with native lysozyme accelerates the refolding of the denatured protein by shifting the equilibrium through binding of the native form [149]. Bossi et al. showed that oxidative refolding of the reduced 25-amino-acid peptide hormone hepcidin is facilitated by binding to an MIP, promoting the formation of disulfide bonds. This behavior is analogous to the “induced fit” mechanism observed in biopolymers [150].

## AI-assisted MIP design and signal processing

The field of molecular imprinting is undergoing a digital transformation, shifting from empirical trial-and-error toward predictive engineering [151]. As target complexity increases—from small molecules to large proteins—the integration of artificial intelligence (AI) and machine learning (ML) enables navigation of the vast polymer design space with unprecedented precision [152–154]. This section explores how data-driven strategies revolutionize rational MIP design (6.1), enhance signal interpretation (6.2), enable edge AI integration (6.3), and address emerging challenges (6.4).

### Current paradigms: from empirical screening to “materiomics”

This shift is best captured by the emerging “Materials 4.0” paradigm, where AI enables navigation of the vast polymer design space [155, 156]. Modern approaches emphasize uncertainty-aware frameworks that quantify prediction reliability [157], establishing in silico process analytical technology (PAT) as a bridge to transform MIP synthesis into a computable engineering discipline [158].

#### AI-driven regression and performance prediction: from micro-scale affinity to macro-scale success

Traditional MIP development relies on resource-intensive iterative screening. The field now shifts toward “materials informatics,” where supervised ML models forecast binding capacity (Q) before synthesis [159]. Özçelik et al. (2026) [159] and Dokuzparmak et al. (2026) [160] demonstrated this with CatBoost and Gaussian process regression (GPR) models predicting Q values for C-reactive protein (CRP) and amphetamine-type stimulants (ATS). Both models were selected through a broader benchmarking exercise against alternative regressors (including Extra Trees and XGBoost), following a GroupKFold cross-validation scheme designed to prevent replicate-based data leakage; Optuna-based hyperparameter optimization was then used to identify the configuration offering the best generalization rather than the lowest training error. CatBoost’s ordered boosting scheme reduces the risk of overfitting on the small, replicate-structured datasets typical of MIP sensor development, while GPR’s non-parametric, kernel-based formulation additionally yields a full predictive distribution rather than a single point estimate—an advantage when guiding costly wet-lab experiments, though at the cost of higher computational demand as dataset size grows. All models were implemented in Python (scikit-learn, CatBoost, and Optuna libraries). To address the small data bottleneck [152], they implemented experimentally constrained data augmentation using deterministic interpolation and stochastic Gaussian sampling, allowing models to learn from chemical distributions rather than limited samples. GPR models provide both predictions and statistical confidence intervals, guiding researchers to optimal design regions. Explainable AI (SHAP analysis) identifies dominant parameters (incubation time, pH, monomer ratios), transforming molecular imprinting into a precise, data-driven discipline [161, 162].

#### Epitope selection via deep learning

Optimal peptide selection remains a bottleneck addressed by computational surface mapping to identify regions with high solvent accessibility and structural stability [163]. For CRP, Özçelik et al. (2026) employed structural mapping followed by CatBoost training on augmented datasets, ensuring chosen epitopes yield polymers with predictable high binding capacity (Q_max = 350 mg/g). Moldovean-Cioroianu et al. (2025) [164] demonstrated hybrid MD-AI approaches for predicting epitope orientation and conformational fidelity at nano-interfaces, ensuring vertical or flat orientations are maintained during synthesis [165], eliminating trial-and-error risks.

#### Nanomaterial-MIP interface modelling: AI-driven surface engineering

Nanomaterial-polymer interfaces require precise control of shell thickness and grafting density. AI-driven interface modelling enables rational hybrid material design [161]. The transition toward sustainable nanotechnology is exemplified by Sezer et al. (2025) [166], who developed green carbon dots (GCDs) with AI-optimized synthesis conditions and predicted optical properties. Arreguin-Campos et al. (2021) [167] demonstrated that meticulously controlled surface imprinting is essential for biomolecular recognition in real matrices. Yarahmadi et al. (2023) [168] showed ensemble learning algorithms accurately predict MIP quality by mapping monomer composition-binding affinity relationships, transforming nano-bio junctions into precisely engineered gateways.

### Signal processing and interpretation: from raw data to explainable insights

Nanomaterials (gold nanoparticles, MXenes, graphene derivatives) enhance electrochemical signals [97], but complex matrices generate non-linear responses requiring AI interpretation [169]. ML transforms raw voltammograms into actionable insights [170].

#### Noise reduction and feature engineering

Complex electrochemical datasets necessitate advanced feature extraction. Peak current density, oxidation/reduction potential shifts, charge-transfer resistance, and double-layer capacitance serve as inputs for ML models like random forest or support vector machines. Software-based digital filtering (Savitzky-Golay, Kalman, Gaussian) improves signal-to-noise ratios without hardware modifications. Özçelik et al. (2026) applied this to detect vitamin E acetate in e-liquids [165], isolating analyte-specific peaks from solvent interferences.

#### Hybrid intelligence and LoD-free quantification

A paradigm shift toward “hybrid intelligence” combines nanopolymeric receptor affinity with AI adaptive learning, addressing stochastic electrochemical signals in complex environments. LoD-free (limit of detection-free) quantification redefines analytical limits. Dokuzparmak et al. (2025) [160] and Sezer et al. (2025) [166] demonstrated that hybrid intelligence internalizes noise profiles—background fluctuations, charging currents, and matrix-induced baseline drifts become informative features fed into CatBoost or Random Forest models. Teaching models to recognize both analyte-induced signals and matrix interference yields superior discriminatory power.

The “Split-and-Conquer” AI architecture for CRP sensing [159] employs physics-informed regional hybrid machine learning, dividing electrochemical responses into concentration regions with specialized algorithms informed by electrochemical kinetics. This bypasses traditional LoD/LoQ constraints, conquering high-noise low-concentration regions through targeted regional learning. In clinical biomarker analysis (CRP in human serum) or vitamins in food matrices, intelligence-enhanced sensing remains robust independent of traditional detection thresholds, critical for food safety [171] and clinical diagnostics [169]. Transforming noise from error source to contextual information ensures next-generation sensor reliability. This approach resonates with a broader shift in ML-aided electrochemical biosensing toward treating matrix noise as an exploitable signal component rather than a nuisance variable to be filtered out [169], suggesting the concept reflects a wider methodological trend rather than an isolated proposal.

#### Multi-analyte discrimination via multi-channel sensor architectures

MIP-based diagnostics evolve toward integrated platforms combining multiple analytes on multi-channel screen-printed carbon electrode (SPCE) arrays. The primary challenge is managing inter-channel cross-talk and signal integrity. AI enables sensor-computing integration through closed-loop hardware-software layers [172]. Liu et al. (2025) [173] showed AI-empowered electrochemical sensors perform real-time data fusion and error correction across sensing nodes through regional calibration and ML algorithms. Multimodal sensing capabilities transition devices to intelligent healthcare tools. Guo et al. (2026) [172] envision seamless multimodal signal integration (electrochemical + physical parameters) through edge-computing architectures. This LoD-free paradigm uses one channel as matrix noise monitor while others perform quantification—the next frontier in decentralized clinical diagnostics [173].

### Toward edge AI-integrated point-of-care platforms

Realizing intelligent healthcare in the field requires sensor-computing integration where data acquisition and computation operate in localized feedback loops [172]: computationally heavy deep learning architectures remain on cloud servers for global model refinement, while lightweight models are embedded directly into POC microcontrollers via TinyML technologies for real-time on-chip classification [174]. The split-and-conquer architecture [159] is well suited to this edge deployment, since partitioning voltammograms into localized kinetic zones reduces the feature space and computational footprint required for low-power processors. Liu et al. (2025) [173] demonstrated that AI-empowered biomedical sensors running on local hardware can perform instantaneous data fusion and autonomous error correction, enabling LoD-free sensing [160] in which one SPCE channel continuously monitors matrix noise while parallel channels quantify the target.

This is not merely a technical convenience: Nangare and Jadhav (2025) [175] noted that rapid, cost-effective, highly selective monitoring of critical biomarkers such as CRP is a genuine clinical need, since delays in detection directly affect patient outcomes. Combining multi-channel MIP sensor arrays with edge-calculated TinyML models is therefore a route toward decentralized diagnostics delivering laboratory-grade quantification from a single drop of whole blood.

### Challenges and future directions

Several challenges must still be resolved before this convergence of MIPs, nanomaterials, and AI translates into routine clinical or field use. On the computational side, power, memory, and latency constraints at the edge still require further algorithmic optimization—weight pruning, network quantization, and knowledge distillation are the most promising routes to compressing split-and-conquer-type models further for deployment on resource-constrained microcontrollers.

A second, arguably more fundamental, challenge is regulatory: the “black-box” nature of many ML models is incompatible with clinical acceptance, since practitioners cannot act on predictions they cannot verify. Embedding XAI frameworks such as SHAP into the analytical validation stage—demonstrating that a model tracks genuine nanopolymer-analyte binding rather than confounding variables such as background noise, temperature, or capacitive currents—will be necessary before AI-assisted MIP sensors can meet clinical validation standards.

Finally, sustainability considerations are increasingly relevant: in silico screening of nano-interface interactions can reduce hazardous reagent use and wet-lab trial-and-error cycles, aligning MIP development with green analytical chemistry principles [176, 177].

Table S4 summarizes the principal computational methods discussed in this section, together with their main applications, advantages, and limitations.

## Conclusions

MIPs exhibit affinities toward low molecular weight analytes, biomacromolecules, and cells that are comparable to those of antibodies—a characteristic that has led to the term plastibodies. For most MIPs, dissociation constants range from the millimolar to the nanomolar regime, with sub-nanomolar and even attomolar detection limits reported for peptide and protein targets. The molecular mechanisms underlying such high affinities—particularly how interactions between a target molecule and a polymer composed of only a few monomers can generate antibody-like affinities, or how imprinted cavities in ion-imprinted polymers discriminate between ions—remain incompletely understood and represent an active area of fundamental research [178]. Addressing these challenges through improved polymer design and a deeper understanding of binding mechanisms will be crucial for the future development and wider commercialization of MIP-based technologies.

MIPs have already achieved commercial success in chromatography and solid-phase extraction and are approaching routine application in analytical measurements for antibiotics, environmental toxins, pharmaceuticals, protein biomarkers, cell typing, and allergen detection. These synthetic binders are produced without laboratory animals and, in contrast to antibodies, can be generated for toxic or weakly immunogenic substances, multi-analyte detection systems, and even structurally unknown targets. Their chemical stability permits use in organic solvents and at elevated temperatures, while their production cost remains substantially lower than that of antibodies. Beyond analytical applications, MIPs are evolving toward controlled release systems: dual-imprinted nanopolymeric structures now enable simultaneous and controlled release of drug combinations such as salmeterol and fluticasone [179].

Despite these advantages, MIPs have thus far achieved only limited commercial penetration in clinical diagnostics. A small but increasing number of companies offer MIP-based materials in various formats such as solid-phase extraction sorbents, affinity resins, and nanoMIP reagents. These products are primarily applicable to purification and bioprocess applications. Challenges that still limit the widespread application of MIPs in analytical chemistry include reproducibility, large-scale manufacturing, standardization, and regulatory and manufacturing challenges associated with the transition toward clinical applications. The high cost and limited availability of protein templates required for the large-scale production of protein MIPs are further challenges [180]. However, the key challenge in real-sample analysis is the typical cross-reactivity of 2–10% toward components of clinical matrices such as human serum albumin and immunoglobulin G, which are present in thousand- to 100,000-fold excess relative to relevant protein biomarkers. This limitation can be mitigated in formats with many “separation plates,” as in columns still used in SPE.

MIPs are increasingly integrated into devices for continuous, minimally invasive, wearable, and implantable sensing. Wearable MIP sensors are a new trend enabling non-invasive real-time measurements in complex body fluids such as saliva, sweat, or tears, thereby monitoring human health status. These on-body sensing systems, such as smart watches, offer insights into personalized physiological state monitoring. Problems still arise from the limited long-term stability of the recognition layer, including nonspecific adsorption and fouling in complex biological matrices, integration with miniaturized transducers and wireless readout systems, calibration, and quantitative performance under continuously changing conditions [178].

The integration of artificial intelligence is transforming this trajectory. What once required months of iterative laboratory optimization—screening monomer combinations, testing polymerization conditions, and characterizing binding properties—can now be accelerated through in silico rational design.

## Supplementary Information

Below is the link to the electronic supplementary material.

ESM 1 (DOCX 58.3 KB)

## Data Availability

The original data are available upon request from the corresponding author.

## Declarations

### Use of generative AI

Generative AI was used to assist in the creation of the schematic illustrations presented in Figs. 6 and 7. The authors reviewed and verified the scientific content and accuracy of the final figures and take full responsibility for their content.
