# Supplementary material for: Analytical applications of molecularly imprinted polymers: a personal view
Source: Anal Bioanal Chem. 2026 Sep 21;418(23):7803–25. doi: 10.1007/s00216-026-06800-y (PMC13744472; doi:10.1007/s00216-026-06800-y)
Supplement: Supplementary file 1 [file 216_2026_6800_MOESM1_ESM.docx]

**Supplementary Material**

**Analytical Applications of Molecularly Imprinted Polymers: A Personal View**

Aysu Yarman^1*^, Aysel Oktay^1,2^, Emine Sezer^3,4^* Sinan Akgol^3,5^, Anne Ettner^6^, Zeynep Sabahat Yunt Ghanbari^1^, Cem Bulent Ustundag^2^, Frieder W. Scheller^7^*

¹ Turkish-German University, Faculty of Science, Molecular Biotechnology, Istanbul 34820, Turkey

² Yildiz Technical University, Faculty of Chemical and Metallurgical Engineering, Department of Bioengineering, Istanbul 34220, Turkey

^3^ Ege University, Biorege Polymeric Nanosystems & AI Assisted Applications Laboratory, Bornova, Izmir 35100, Türkiye

^4^ Ege University, Faculty of Computer and Information Sciences, Department of Computer Engineering, Bornova, Izmir 35100, Türkiye

^5^ Ege University, Faculty of Science, Department of Biochemistry, Bornova, Izmir 35100, Türkiye

^6^ Anne Ettner Pharma Consulting/Wollbach 79400, Germany

^7^ University of Potsdam, Institute of Biochemistry and Biology, Karl-Liebknecht-Strasse 24-25, Potsdam 14476, Germany

[*](mailto:*yarman@tau.edu.tr) [fschell@uni-potsdam.de](mailto:fschell@uni-potsdam.de), [yarman@tau.edu.tr](mailto:yarman@tau.edu.tr), emine.sezer@ege.edu.tr

**Table S1.** Comparative overview of protein MIPs prepared by epitope approaches**.**

| Template | MIP structure | Epitope/Peptide | Selectivity | Linear measuring range | IF and K_d_ | Ref. |
| --- | --- | --- | --- | --- | --- | --- |
| HbA | Surface epitope-imprinted polymer particles (MAA/Am/EGDMA-based polymer synthesized on silica nanoparticles) | VLSPADK (α-chain), VHLTPEEK (β-chain) | Recombinant HbF: low selectivity  HbA-haptoglobin (Hp), BSA, myoglobin: high selectivity | — | IF = ~ 1.7 (at pH 7.4 for HbA (FeIII)) | [1] |
| Insulin | *o*-PD as functional monomer, on top of a SAM covered Au electrode | C-insulin polypeptide (C-terminal peptide of insulin) | hIg, BSA, HSA, Con A, L-proline, L-tryptophan | 10 - 500 fM | — | [2] |
| Cyt c, ADH, BSA | Photopolymerization of MIP on a glass or silicon surface | C-terminal nona-peptides of each protein | Exchange of one amino acid | 0 - 289 nM for Cyt c | K_d_ = 72.3 nM for  Cyt c,  IF = ~7 for Cyt c | [3] |
| Cyt c | EP of scopoletin on a submonomolecular layer on gold | Cys-extended C-terminal nona-peptide | BSA, myoglobin: low lysozyme: high due to electrostatic interaction | 0 - 10 μM for Cyt c | K_d_ = 8.54 μM,  IF = 6 for Cyt c,  K_d_ = 2.51 μM, IF = 10 for epitope | [4] |
| Cyt c | Carbon dots/SiO_2_/MIP | C- und N-terminal nona-peptides | Lysozyme, trypsin | 0.1- 40 μM for Cyt c  LOD = 89 nM. | IF = 5.65 for Cyt c | [5] |
| Trf | Fe₃O₄@PES epitope-imprinted beads | MRLAVGALL (N-terminal transferrin epitope) | Selectivity against ribonuclease B, cyt c and β-lactoglobulin | — | Recognition coef. = 3.36 for Trf,  recognition coef. = 4.70 for epitope | [6] |
| HSA, IgG, Trf | Phase inversion-based PES self-assembly | N-terminal peptides | Capture proteins from human plasma | — | Recognition coef. = 2.29, 9.41, and 7.54 for HSA, IgG and Trf | [7] |
| HSA, Trf | Dual-template imprinted polymer on magnetic carbon nanotubes | C-terminal peptides | BSA, Mb, and Cyt c | 0 - 7.5 μM for HSA  0 - 6.3 μM for Trf | IF = 2.57 for HSA  IF = 2.17 for Trf | [8] |
| NSE | EP of scopoletin on a SAM on gold electrode (template immobilized via cysteine-gold interaction, template removal by anodic desorption at 0.9 V) | Cys-extended helical peptide (LKAVDHINST, 10-mer), computationally selected stable α-helical epitope of NSE | High selectivity against Ep4, Ep5, and BSA | Ep1 peptide: 2–500 µM  NSE protein: 0.5–100 ng mL⁻¹ | IF = 4.6,  K_d_ = 12.2 µM for peptide,  K_d_ = 53 pM for NSE | [9] |
| IgG | Solid-phase synthesized nanoMIPs; epitope immobilized on glass beads via cysteine–maleimide chemistry; monomers: NIPAm, TBAm, acrylic acid | Fc-domain C-terminal epitope peptide: QKSLSLSPGK | Selective recognition of both human IgG and goat IgG, mimicking protein A-like Fc binding | 0.02–0.67 nM | K_d_ = 2.5 nM for peptide, K_d_ = 27.4 nM for protein | [10] |
| Oxytocin | EP of thiophene-based functional monomers on gold integrated with a microfluidic capacitive sensor | Whole oxytocin nonapeptide (Cys-Tyr-Ile-Gln-Asn-Cys-Pro-Leu-Gly-NH₂) directly imprinted | High selectivity against carbetocin (close analog) and smaller amino acid interferents/metabolites | 0.06 – 1 mM | IF = 22 | [11] |
| Cyt c | Microemulsion polymerization of 3-aminopropyltriethoxysilane | C-terminal octapeptide epitope: YLKKATNE; optimized template: palmitic acid-modified epitope (16C-PAL-YLKKATNE) for oriented imprinting | Selective against myoglobin, horseradish peroxidase, human serum albumin | 0.40 – 4.0 nM | IF = 3.0 | [12] |
| L-thyroxine (T4) | Core–shell magnetic MIP (Fe₃O₄@SiO₂ based magnetic-MIP); experimentally compared monomers: itaconic acid, acrylic acid, acrylamide (final optimized MIP: itaconic acid) | — | Selective against common serum interferents (uric acid, ascorbic acid, glucose, dopamine) | 3.88 × 10⁻⁶ – 388.4 µg mL⁻¹ | — | [13] |
| ß2-Microglobulin | Polycondensation of multiple silylating reagents: APTES/UPTES/IBTES/TEOS | C-terminus nonapeptide | RNase A, RNase B, HRP, and BSA |  | IF = 5.8  K_d_ = 0.2 µM for peptide | [14] |
| HSA | Self-polymerization of dopamine on Ni^2+^ modified magnetic nanoparticles | His-tag modified C-terminal peptide | BSA and Cyt c |  | IF = 4.26 for epitope  IF = 2.12 for HSA | [15] |
| HSA | MIP on modified SiO_2_ NPs | His-tag-anchored epitope | BSA, myoglobin, ribonuclease B | 9 - 15 μM for HSA | IF = 4.0 for HSA | [16] |
| His-tagged GFP | Epitope imprinting enhanced IMAC: SiO₂@IDA/MAPS-Ni²⁺ nanoparticles coated with thin surface-imprinted polymer shell via RAFT polymerization; functional monomer: acrylamide | His-tag epitope (HHHHHH) | Adsorption toward His-tagged GFP was ~6× higher than NI-IMAC; purified product purity improved to 52.9% vs 48.0% for conventional IMAC | — | IF = 7.1; K_a_ (high-affinity sites) = 2.318 × 10³ M⁻¹; K_a_ (low-affinity sites) = 0.053 × 10³ M⁻¹ | [17] |
| A neural cell adhesion molecule (CD56) | EP of p-aminobenzeneboronic | Polysialic acid (PolySia) | Selective against CA19-9, PSA, BSA, bovine hemoglobin, glucose, ascorbic acid, uric acid | 10–150 ng/L | — | [18] |
| Carbohydrate antigen 19-9 | EP of *o*-PD on a GCE | SLe^a^ polysaccharide glycan epitope and NeuAc monosaccharide glycan epitope | NCAM-1, BSA, BHb, ConA, D-man, glucose, DL-Cys, Vc, uric acid | 1–50 U/mL for monosaccharide,  0.1–5 U/mL for polysaccharide | — | [19] |
| NMP22 | Electropolymerization of o-PD on G-SH/AuNPs/AuE; EMIP film with overoxidation elution | C-terminal dodecapeptide (GATPRAKGKAKH) | AFP, HSA, CEA, PSA: high selectivity (IF = 15.93 for NMP22) | 0.1 pg mL⁻¹ – 1.0 µg mL⁻¹; LOD = 0.077 pg mL⁻¹ | IF = 15.93 for NMP22 | [20] |
| SARS-CoV-2 spike RBD | EP of Asp-UMB on gold SPRi chip; MIP microarray format | Heptapeptide GFNCYFP from RBD | HSA: high selectivity; CGGGH (random peptide): low binding | 1–86 nM for RBD | K_d_ = 30 pM for RBD | [21] |
| hCG | Free radical polymerization of NIPAm/TBAm/AA/APM/BIS on magnetic template carriers; dispersed phase epitope imprinting | SV: β-chain loop aa 66–80 (SIRLPGCPRGVNPVV); PQ: C-terminal β-chain aa 135–145 (PGPSDTPILPQ) | LH: high selectivity (structurally similar hormone); BSA: high selectivity | 4–64 nM (SPR kinetic range); QCM: 12.5–100 µg mL⁻¹ | K_d_ = 85 nM (MIP-PQ, SPR); K_d_ = 92 nM (MIP-SV, SPR); K_d_ = 8.9 nM (MIP-PQ, QCM); K_d_ = 4.4 nM for hCG (MIP-PQ, QCM) | [22] |
| sST2 | EP of poly(AN-co-MSAN) on ITO electrodes doped with Mo₃C₂ MXene; EG-FET platform | ɕGEDYYSVENPɤ (pGE) — surface-exposed loop of sST2 | Albumin, lysozyme: high selectivity; serum samples | 1.0 fg mL⁻¹ – 100.0 pg mL⁻¹; LOD = 0.05 fg mL⁻¹ | — | [23] |
| DJ-1 | EP of poly(AN-co-MSAN) on ITO electrode (AN:MSAN = 1:1) | AKDPVQCSRDVVICPDU (pK) — surface-exposed loop (aa 41–55) | HSA, lysozyme, SNCA, lysozyme, ascorbic acid, uric acid | 1.0×10⁻¹² – 1.0×10⁻⁸ g L⁻¹; LOD = 1.0×10⁻¹³ g L⁻¹ | IF = ~1.87 | [24] |
| Dengue NS1 | Precipitation polymerization on solid-phase synthesis; MIP nanoparticles (NIPAm/BIS/AAc); AuSPE; competitive and direct DPV assays | Ac-VHTWTEQYKFQ-CONH₂ — conserved loop; recognized by mAb 4F6 | HSA, BSA, FbG: high selectivity; spiked human serum | Competitive: epitope 1–100 ng mL⁻¹, NS1 5–300 ng mL⁻¹; LOD = 0.74 / 4.30 ng mL⁻¹. Direct: epitope 5–100 ng mL⁻¹, NS1 10–100 ng mL⁻¹; LOD = 3.82 / 8.23 ng mL⁻¹ | IF = ~5 (imprinting factor vs. reference NPs) | [25] |
| PSA | EP of *o*-PD on AuNPs/AuE (β-MEA-anchored); EMIP film | KWIKDTIVANP (C-terminal PSA epitope) | BSA, HSA, AFP, CEA: high selectivity; human serum (recovery 95.9–106.0%) | 1.0 fg mL⁻¹ – 0.1 μg mL⁻¹; LOD = 0.31 fg mL⁻¹ | IF = ~5.4 | [26] |
| Lactoferrin | Bulk polymerization (MPC/BMA/4-ATP/EGDMA) followed by electrodeposition on Au-EQCM; SAM@AuNPs as template carrier; zwitterionic matrix | Lactoferricin B (FF-25; FKCRRWQWRMKKLGAPSITCVRRAF) — N-terminal region (selected by molecular docking) | Structurally analogous peptides: high selectivity; plasma proteins (globulin, albumin, thyroglobulin, transferrin): high selectivity; real milk samples (cow, buffalo, goat) | 100–700 nM; LOD = 5.25 nM; LOQ = 17.51 nM | IF = 10.91 | [27] |
| Strep-tag II peptide (Cys-Strep) | Hierarchical electropolymerized polySP nanofilm on Au electrode; first chemisorption of Cys-Strep on Au via thiol-gold interaction, followed by electropolymerization of scopoletin | WSHPQFEK-C (cysteine-extended Strep-tag II peptide) | High selectivity toward Strep-tagged proteins (Strep-MBH and Twin-Strep-ALP); negligible binding observed for His6-tagged MBH, and significantly reduced binding for scrambled/modified peptide sequences | — | Apparent K_d_ = 2.54 nM for peptide; Apparent K_d_ = 34.85 nM for Strep-MBH protein | [28] |
| Full-length CD44 glycoprotein + CD44 epitope peptide (sequential imprinting) | Sequential dual-MIP system (SEPI): first P-PMIP (Fe₃O₄-EDTA/Cu²⁺ magnetic epitope-imprinted silica nanoparticles) → then E-MIP (Au@CB/PLA electrode coated with polydopamine protein-imprinted layer) | His-tagged CD44 epitope peptide: HHHHHHPIRDTSDT | High selectivity against HER2, CD47, PDL1, Gal3, CD73 (for P-PMIP stage); final sensor also showed high selectivity against AFP, CEA, PSA, BSA with <10% response even at 100-fold higher concentration | 0.5 pg mL⁻¹ – 200 ng mL⁻¹  LOD = 9.98 fg mL⁻¹ | IF_peptide_ = 4.97 IF_sensor_ = 3.72 | [29] |

**Abbreviations:** HbA: adult human hemoglobin A; MAA: methacrylic acid; Am: acrylamide; EGDMA: ethylene glycol dimethacrylate; HbF: fetal human hemoglobin; hCG: human chorionic gonadotropin; *o*-PD: *o-*phenylenediamine; SAM: self-assembly monolayer; Au: gold; hIg: human immunoglobulin; BSA: bovine serum albumin; HSA: human serum albumin; Con A: concanavalin A; Cyt c: cytochrome c; ADH: alcohol dehydrogenase; EP: electropolymerization; Cys: cysteine; Trf: transferrin; PES: polyethersulfone; IgG: Immunoglobulin G; Mb: myoglobin; NSE: neuron specific enolase; RNase: ribonuclease; His: histidine; GFP: green fluorescent protein; IMAC: metal ion affinity chromatography; IDA: iminodiacetic acid; MAPS: 3-(trimethoxysilyl)propyl methacrylate; RAFT: reversible addition-fragmentation chain transfer; SLe^a^: 3′-Sialyl Lewis A tetrasaccharide; NeuAc: N-acetylneuraminic acid; NCAM-1: neural cell adhesion molecule-1; BHb: bovine hemoglobin; D-man: D-mannose; DL-Cys: DL-cysteine; Vc: ascorbic acid; GCE: glassy carbon electrode; G-SH: thiolated graphene; AuNPs: gold nanoparticles; AuE: gold electrode; EMIP: epitope molecularly imprinted polymer; AFP: alpha fetoprotein; CEA: carcinoembryonic antigen; PSA: prostate-specific antigen; RBD: receptor binding domain; Asp-UMB: aspartate-umbelliferone conjugate; SPRi: surface plasmon resonance imaging; hCG: human chorionic gonadotropin; NIPAm; N-isopropylacrylamide; TBAm: N-tert-butylacrylamide; AA: acrylic acid; APM: N-(3-aminopropyl)methacrylamide hydrochloride; BIS: N,N’-methylenebisacrylamide; LH: lutenizing hormone; QCM: quartz crystal microbalance; AN: aniline; MSAN: aminobenzenesulfonic acid; EG-FET: extended gate in a field-effect transistor; ITO: indium tin oxide; SNCA: α-synuclein; AAc: acrylic acid; AuSPE: screen-printed gold electrode; DPV: differential pulse voltammetry; mAb: monoclonal antibody; FbG: fibrinogen, β-MEA: β-mercaptoethylamine; MPC: 2-methacryloyloxyethyl phosphorylcholine; BMA: benzyl methacrylate; 4-ATP: 4-aminothiophenol; EQCM: electrochemical quartz crystal microbalance; polySP: polyscopoletin.

**The authors previously reviewed MIP sensing concepts and representative applications for drugs and protein biomarkers [30]. Building on that published work, Table S2 provides a nanomaterial-focused synthesis and incorporates recent examples to emphasize how the material component contributes to analytical performance.**

**Table S2. Representative nanomaterials integrated into MIP-based sensing platforms and their principal analytical advantages. The selection builds on the authors’ previous review [30] and is updated with recent studies.**

| Nanomaterial | Sensing platform | Transduction method | Target analyte | Principal analytical advantage | Ref. |
| --- | --- | --- | --- | --- | --- |
| N-doped graphene oxide nanoribbons/Au-Pt bimetallic NPs | MIP-modified glassy carbon electrode | Electrochemical; differential pulse voltammetry (DPV) | Perfluorooctane sulfonate (PFOS) | Combines a high-area conductive support with Au-Pt electrocatalysis, increasing charge transfer and enabling ultrasensitive detection in water. | [31] |
| Functionalized Au nanoparticles (AuNPs) | AuNP/MIP-modified screen-printed carbon electrode | Electrochemical; DPV | Caffeine | Thiol-compatible surface functionalization supports stable MIP integration; AuNPs enlarge the electroactive area and improve electron transfer for sensitive quantification. | [32] |
| Ag nanoparticles (AgNPs) | AgNP-doped surface-imprinted polymer | Surface-enhanced Raman scattering (SERS) | Circulating tumor cells | Strong plasmonic enhancement provides molecular fingerprinting and high signal amplification, while surface imprinting supplies cell selectivity. | [33] |
| Pt nanoparticles (PtNPs) | Pt-modified screen-printed carbon electrode integrated with MIP | Electrochemical sensing | Ethanol | Pt electrocatalysis accelerates ethanol oxidation and increases current response; the disposable printed platform supports selective, low-volume analysis. | [34] |
| Carbon quantum dots/TiO2 | CQD-TiO_2_ photoactive substrate coated with MIP | Photoelectrochemical | Triazophos | The CQD/TiO_2_ heterostructure improves light harvesting and charge separation; imprinting adds pesticide selectivity for water analysis. | [35] |
| ZnO nanoparticles | ZnO-assisted MIP electrochemical sensor | Electrochemical; voltammetric | Valganciclovir | High surface area and semiconducting ZnO promote analyte accumulation and interfacial electron transfer, supporting sensitive and selective pharmaceutical analysis. | [36] |
| Reduced graphene oxide (rGO) | rGO nanosheet/MIP-modified electrode | Electrochemical; voltammetric | p-Cresyl sulfate | Restores conductive pathways beneath the imprinted film and increases electroactive area, improving sensitivity toward a clinically relevant uremic toxin. | [37] |
| rGO/polyaniline-poly(o-phenylenediamine) composite | Composite MIP-modified electrode | Electrochemical; DPV | Ciprofloxacin | The conductive rGO/polymer interface enhances current response and film stability; molecular cavities enable selective antibiotic determination. | [38] |
| MIP nanoparticles/carbon nanotubes (CNTs) | MIP-NP/CNT chemiresistive gas-sensing composite | Electrical resistance | Methanol vapor | The percolating CNT network converts vapor rebinding into a rapid resistance change, while nanoMIPs provide selective recognition in the gas phase. | [39] |
| Single-walled carbon nanotubes (SWCNTs) | MIP/SWCNT-modified glassy carbon electrode | Electrochemical; voltammetric | Zineb | SWCNTs increase conductivity and accessible surface area, improving signal magnitude and enabling fungicide determination in food samples. | [40] |
| Laser-induced graphene (LIG) | Flexible LIG electrode with electropolymerized MIP | Electrochemical | Perfluorooctanoic acid (PFOA) | A binder-free, high-area conductive architecture supports miniaturization and ultratrace PFAS detection (reported LOD 0.025 ppt) with discrimination against related PFAS. | [41] |
| Graphene quantum dots/AuNPs | GQD-AuNP composite MIP electrode | Electrochemical; voltammetric | 17β-Estradiol | The hybrid combines rapid electron transfer and a large functional surface with MIP selectivity, increasing sensitivity for hormone determination. | [42] |
| Cu-BTC MOF/graphene | Cu-BTC/graphene composite MIP electrode | Electrochemical; voltammetric | Chloramphenicol | MOF porosity concentrates the analyte and exposes more imprinted sites, while graphene supplies conductivity for sensitive antibiotic detection. | [43] |
| Cu-MOF/rGO/AuNPs | MIP/Cu-MOF/rGO/AuNP-modified glassy carbon electrode | Electrochemical | Electroneutral organophosphorus pesticides | The multicomponent interface couples MOF enrichment with rGO/AuNP charge transfer, overcoming the weak electroactivity of electroneutral pesticide targets. | [44] |
| Amphiphilic block copolymer/MOF | MOF-based fluorescent MIP sensor | Fluorescence | Oxytetracycline | The porous fluorescent scaffold offers abundant accessible sites and signal amplification; the amphiphilic environment supports analysis in milk. | [45] |
| Fe-MOF-NH_2_/CNT-NH_2_/MXene | Ratiometric MIP electrochemical platform | Ratiometric electrochemical | Ofloxacin | Multiple conductive and porous components provide internal signal referencing, high site accessibility, and improved robustness for antibiotic quantification. | [46] |
| Eu-BCA MOF | Eu-BCA MOF coated with MIP | Fluorescence | Zearalenone | Eu-based luminescence enables sensitive optical readout, while the MIP shell suppresses interference and supports selective mycotoxin detection in corn oil. | [47] |
| TiO_2_@Ti_3_C_2_Tx MXene | TiO_2_@Ti_3_C_2_Tx/MIP-modified electrode | Electrochemical; DPV | Chlortetracycline | The conductive, hydrophilic MXene provides a large accessible interface; TiO_2_ contributes adsorption and interfacial synergy for sensitive antibiotic detection. | [48] |
| Ti_3_C_2_Tx MXene/graphene | MXene/graphene composite MIP electrode | Electrochemical | Cortisol | The two-dimensional hybrid limits sheet restacking, preserves conductive pathways, and increases accessible imprint sites for ultrasensitive biomarker detection. | [49] |
| Amine-functionalized multi-walled carbon nanotubes (MWCNT-NH_2_) | Photopolymerized ACR-ABC/MWCNT-NH_2_@MIP on a glassy carbon electrode | Electrochemical; voltammetric/impedimetric | Abacavir | MWCNT-NH_2_ increases active surface area, porosity and binding-site accessibility. The platform enabled femtomolar detection in standard and serum samples, with low-cost and green-analysis credentials. | [50] |
| Reduced graphene oxide (rGO) | Electropolymerized poly(o-aminophenol) MIP/rGO on a glassy carbon electrode | Electrochemical; DPV | Paracetamol | rGO improves the conductive interface and analytical response. The reusable sensor achieved a 10 nM LOD, 20 reuse cycles, 15-day stability and agreement with HPLC in pharmaceutical samples. | [51] |

**Abbreviations: ABC: abacavir; ACR: acrylamide; AuNPs: gold nanoparticles; CNTs: carbon nanotubes; CQD: carbon quantum dot; DPV: differential pulse voltammetry; GCE: glassy carbon electrode; HPLC: high-performance liquid chromatography; LIG: laser-induced graphene; MIP: molecularly imprinted polymer; MOF: metal-organic framework; MWCNT-NH2: amine-functionalized multi-walled carbon nanotubes; NPs: nanoparticles; PFAS: per- and polyfluoroalkyl substances; PFOS: perfluorooctane sulfonate; PFOA: perfluorooctanoic acid; rGO: reduced graphene oxide; SERS: surface-enhanced Raman scattering; SPCE: screen-printed carbon electrode; SWCNTs: single-walled carbon nanotubes.**

**Table S3.** Representative examples (since 2020) of synergistic integration of enzymes and enzyme-mimicking systems with MIP-based electrochemical sensors.

| Template/ Analyte | Functional Monomer (with or without crosslinkers) | Electrode | Detection Method | Measuring Range & LOD | Ref. |
| --- | --- | --- | --- | --- | --- |
| HRP | β-Cyclodextrin (β-CD) | GCE | DPV; Redox Marker +  Chronoamperometry (H₂O₂) | 10 ng/mL–0.1 mg/mL  LOD: 2.23 ng/mL  H₂O₂: 1–15 µM, LOD 0.4 µM | [52] |
| Pyruvate Oxidase | 4-aminophenol | PtNP-PME | CV; Catalytic Activity | 25–700 U/L  LOD: 2.97 U/L | [53] |
| α-Amylase | Pyrrole | AuSPE | SWV; Redox Marker | 3.0 × 10^−4^ -0.60 mg mL^−1^ in buffer solution; 3.0 × 10^−4^ -3.0 × 10^−2^ mg mL^−1^ in human saliva LOD < 3.0 × 10^−4^ mg mL^−1^ | [54] |
| Lysozyme | Scopoletin | Au electrode | EIS; Redox Marker | Up to 20 µM  LOD: 62 nM and 141 nM | [55] |
| Lysozyme | Pyrrole | GO@Fe₃O₄-Au electrode | EIS; Redox Marker | 1 pg/mL- 0.1 µg/mL  LOD: 0.009 pg/mL | [56] |
| Lysozyme | Mixture (NIPAm / tBuAm / APMA / AA) | Graphite SPE | EIS; Redox Marker | LOD: 13 pM | [57] |
| Trypsin | *o*-PD | Porous Au-QCE | EIS; Redox Marker | hs-MIP: 0.24–24 µg/mL | [58] |
| Trypsin | Mixture [BIS:N,N′-methylenebisacrylamide, EGDMA: Ethylene glycol dimethacrylate, methacrylic  acid, a crylamide, tert‑butyl acrylate, and APMA:N-(3-aminopropyl)methacrylamide hydrochloride (APMA)] | TA-SAM-nanoMIP-SPAuE | EIS; Redox Marker | 3–27 ng/mL  LOD: 1.06 ng/mL | [59] |
| Acid phosphatase | *o*-PD | Chitosan-modified GCE | DPV; Redox Marker | 0.5–100 U/L  LOD: 0.1 U/L | [60] |
| Cys-Strep-tag II peptide  → recognizes Strep-tagged recombinant enzymes | Scopoleten | Au electrode | CV and Amperometry (catalytic currents of bound enzymes) | [NiFe]-Hydrogenase: H₂ oxidation current, CV  ALP: pAPP→pAP substrate conversion current, Amperometry | [28] |
| Cortisol | Pyrrole | CuPcTS /Screen-printed carbon electrode | DPV; Electrocatalytic reduction (ketone→alcohol) | 1 pM – 1 µM  LOD: 181 pM | [61] |
| Carbendazim | *o*-PD | AuNP-rGO/GCE | DPV; GOx-mediated  ferrocene recycling | 2.0×10⁻⁹–7.0×10⁻⁵ M  LOD: 0.68 nM | [62] |

**Abbreviations:** HRP: horseradish peroxidase; β-CD: beta-cyclodextrin; GCE: glassy carbon electrode; DPV: differential pulse voltammetry; H₂O₂: hydrogen peroxide; PtNP: platinum nanoparticles; PME: platinum microelectrode; CV: cyclic voltammetry; AuSPE: gold screen-printed electrode; SWV: square wave voltammetry; Au: gold; EIS: electrochemical impedance spectroscopy; GO: graphene oxide; NIPAm: N-isopropylacrylamide; tBuAm: tert-butylacrylamide; APMA: N-(3-aminopropyl)methacrylamide; AA: acrylic acid; SPE: screen-printed electrode; *o*-PD: *o*-phenylenediamine; Au-QCE: gold quartz crystal electrode; hs-MIP: hierarchical surface molecularly imprinted polymer; BIS: N,N′-methylenebisacrylamide; EGDMA: ethylene glycol dimethacrylate; TA: thioctic acid; SAM: self-assembled monolayer; SPAuE: screen-printed gold electrode; Cys: cysteine; ALP: alkaline phosphatase; pAPP: *p*-aminophenyl phosphate; pAP: *p*-aminophenol; CuPcTS: copper phthalocyanine tetrasulfonate; AuNP: gold nanoparticles; rGO: reduced graphene oxide; GOx: glucose oxidase.

**Table S4.** Computational methods discussed in Section 6, their applications, and principal advantages/limitations.

| Method | Task | Application | Key advantage | Key limitation | Ref. |
| --- | --- | --- | --- | --- | --- |
| CatBoost | Regression — binding capacity (Q) prediction before synthesis | CRP | Ordered boosting resists overfitting on small, replicate-structured datasets | Requires careful hyperparameter tuning; limited interpretability without SHAP | [63] |
| Gaussian Process Regression | Regression — Q prediction with uncertainty quantification | ATS | Yields full predictive distribution, not just a point estimate; guides costly experiments | Computational cost scales poorly with dataset size; sensitive to kernel choice | [64] |
| CatBoost / Random Forest ("Hybrid Intelligence") | Noise-aware quantification, LoD-free sensing | CRP, ATS | Treats matrix noise as an informative feature rather than an error source | Needs sufficiently large augmented data to learn noise structure reliably | [64, 65] |
| Random Forest / SVM | Feature extraction, noise reduction from voltammograms | General electrochemical signals | Complements digital filtering for signal-to-noise improvement | Performance depends on quality of hand-engineered features | [66] |
| Ensemble / non-linear regression | Predicting imprinting quality from monomer-composition data | General MIP quality prediction | External validation of the approach across an independent group | Ensemble complexity trades off against interpretability | [67] |
| SHAP (explainable AI) | Post-hoc feature attribution | Applied across the models above | Identifies dominant experimental parameters; supports regulatory/XAI needs | Explanation quality depends on the underlying model's faithfulness | [68, 69] |
| TinyML / edge-deployed models | On-chip real-time classification | Multi-channel SPCE point-of-care arrays | Enables decentralized, real-time diagnostics without cloud dependency | Requires aggressive compression; constrained by microcontroller memory/power | [70-72] |

**References**

[1]	H. Bagán, T. Zhou, N.L. Eriksson, et al., Synthesis and characterization of epitope-imprinted polymers for purification of human hemoglobin, RSC Adv. 7 (66) (2017) 41705–41712. https://doi.org/10.1039/C7RA07674F

[2]	C. Zhao, X. Ma, J. Li, An Insulin Molecularly Imprinted Electrochemical Sensor Based on Epitope Imprinting, Chin. J. Anal. Chem. 45 (9) (2017) 1360–1366. https://doi.org/10.1016/S1872-2040(17)61039-9

[3]	H. Nishino, C.-S. Huang, K.J. Shea, Selective Protein Capture by Epitope Imprinting, Angew. Chem. Int. Ed. 45 (15) (2006) 2392–2396. https://doi.org/10.1002/anie.200503760

[4]	D. Dechtrirat, K.J. Jetzschmann, W.F.M. Stöcklein, et al., Protein Rebinding to a Surface-Confined Imprint, Adv. Funct. Mater. 22 (24) (2012) 5231–5237. https://doi.org/10.1002/adfm.201201328

[5]	D.Y. Li, X.M. Zhang, Y.J. Yan, et al., Thermo-sensitive imprinted polymer embedded carbon dots using epitope approach, Biosens. Bioelectron. 79 (2016) 187–192. https://doi.org/10.1016/J.BIOS.2015.12.016

[6]	K. Yang, J. Liu, S. Li, et al., Epitope imprinted polyethersulfone beads by self-assembly for target protein capture from the plasma proteome, Chem. Commun. 50 (67) (2014) 9521–9524. https://doi.org/10.1039/C4CC03428G

[7]	K. Yang, S. Li, J. Liu, et al., Multiepitope Templates Imprinted Particles for the Simultaneous Capture of Various Target Proteins, Anal. Chem. 88 (11) (2016) 5621–5625. https://doi.org/10.1021/acs.analchem.6b01247

[8]	Y.P. Qin, C. Jia, X.W. He, et al., Thermosensitive metal chelation dual-template epitope imprinting polymer using distillation–precipitation polymerization for simultaneous recognition of human serum albumin and transferrin, ACS Appl. Mater. Interfaces 10 (10) (2018) 9060–9068. https://doi.org/10.1021/acsami.8b00327

[9]	Z. Altintas, A. Takiden, T. Utesch, et al., Integrated approaches toward high‐affinity artificial protein binders obtained via computationally simulated epitopes for protein recognition, Adv. Funct. Mater. 29 (15) (2019) 1807332. https://doi.org/10.1002/adfm.201807332

[10]	E. Moczko, A. Guerreiro, C. Cáceres, et al., Epitope approach in molecular imprinting of antibodies, J. Chromatogr. B Anal. Technol. Biomed. Life Sci. 1124 (2019) 1–6. https://doi.org/10.1016/j.jchromb.2019.05.024

[11]	P.S. Sharma, Z. Iskierko, K. Noworyta, et al., Synthesis and application of a “plastic antibody” in electrochemical microfluidic platform for oxytocin determination, Biosens. Bioelectron. 100 (2018) 251–258. https://doi.org/10.1016/J.BIOS.2017.09.009

[12]	X.T. Ma, X.W. He, W.Y. Li, et al., Oriented surface epitope imprinted polymer-based quartz crystal microbalance sensor for cytochrome c, Talanta 191 (2019) 222–228. https://doi.org/10.1016/j.talanta.2018.08.079

[13]	S.L. Moura, L.M. Fajardo, L. dos Anjos Cunha, et al., Theoretical and experimental study for the biomimetic recognition of levothyroxine hormone on magnetic molecularly imprinted polymer, Biosens. Bioelectron. 107 (2018) 203–210. https://doi.org/10.1016/j.bios.2018.01.028

[14]	R. Xing, Y. Ma, Y. Wang, et al., Specific recognition of proteins and peptides via controllable oriented surface imprinting of boronate affinity-anchored epitopes, Chem. Sci. 10 (6) (2019) 1831–1835. https://doi.org/10.1039/c8sc04169e

[15]	S. Li, K. Yang, J. Liu, et al., Surface-Imprinted Nanoparticles Prepared with a His-Tag-Anchored Epitope as the Template, Anal. Chem. 87 (9) (2015) 4617–4620. https://doi.org/10.1021/ac5047246

[16]	S. Li, K. Yang, N. Deng, et al., Thermoresponsive epitope surface-imprinted nanoparticles for specific capture and release of target protein from human plasma, ACS Appl. Mater. Interfaces 8 (9) (2016) 5747–5751. https://doi.org/10.1021/acsami.5b11415

[17]	S. Li, K. Yang, B. Zhao, et al., Epitope imprinting enhanced IMAC (EI-IMAC) for highly selective purification of His-tagged protein, J. Mater. Chem. B 4 (11) (2016) 1960–1967. https://doi.org/10.1039/C5TB02505B

[18]	X. Ma, M. Li, P. Tong, et al., A strategy for construction of highly sensitive glycosyl imprinted electrochemical sensor based on sandwich-like multiple signal enhancement and determination of neural cell adhesion molecule, Biosens. Bioelectron. 156 (2020) 112150. https://doi.org/10.1016/j.bios.2020.112150

[19]	J. Li, X. Ma, M. Li, et al., Does polysaccharide is an idea template selection for glycosyl imprinting?, Biosens. Bioelectron. 99 (2018) 438–442. https://doi.org/10.1016/j.bios.2017.08.004

[20]	X. He, Y. Zheng, Y. Zhang, et al., Highly sensitive and selective determination of nuclear matrix protein 22 based on an epitope imprinted electrochemical biosensor, Sens. Actuators B Chem. (2026) 139623. https://doi.org/10.1016/j.snb.2026.139623

[21]	Z. Bognár, J. Kozma, N. Kovács, et al., Novel functional monomer for the electrochemical synthesis of highly affine epitope-imprinted polymers, Electroanalysis 35 (6) (2023). https://doi.org/10.1002/elan.202300025

[22]	R. Zubrytė, L. Mavliutova, Y. García, et al., Development of molecularly imprinted polymers for the detection of human chorionic gonadotropin, Sci. Rep. 15 (1) (2025) 10436. https://doi.org/10.1038/s41598-025-94289-3

[23]	M.H. Lee, K.H. Liu, J.L. Thomas, et al., Electrosynthesis of molybdenum carbide-doped epitope-imprinted conductive polymers for the determination of soluble suppression of tumorigenicity 2 with a field effect transistor platform, Biosens. Bioelectron. 278 (2025) 117353. https://doi.org/10.1016/j.bios.2025.117353

[24]	M.H. Lee, J.L. Thomas, J.C. Ciou, et al., Synthesis of epitope-imprinted polymers for the electrochemical sensing of protein Deglycase DJ-1 in serum, Microchem. J. 225 (2026) 117955. https://doi.org/10.1016/j.microc.2026.117955

[25]	K. Sirivibulkovit, J. Voelkle, D.J. Windisch, et al., Electrochemical dengue sensor based on NS1 epitope-imprinted polymers, Biosens. Bioelectron. 289 (2025) 117915. https://doi.org/10.1016/j.bios.2025.117915

[26]	H. Shao, L. Zhao, Z. Liu, et al., An epitope imprinted electrochemical sensor for highly sensitive and selective determination of prostate-specific antigen, Microchim. Acta 191 (11) (2024) 660. https://doi.org/10.1007/s00604-024-06737-y

[27]	A. Srivastava, R. Raghuwanshi, M. Singh, Computational designing and synthesis of epitope imprinted sensor with zwitterionic polymeric matrix for sensitive, specific and selective sensing of protein, Microchem. J. 212 (2025) 113324. <https://doi.org/10.1016/j.microc.2025.113324>

[28]	A. Yarman, A.F. Waffo, S. Katz, et al., A Strep‐Tag Imprinted Polymer Platform for Heterogenous Bio (electro) catalysis, Angew. Chem. Int. Ed. 63 (47) (2024) e202408979. https://doi.org/10.1002/anie.202408979

[29]	C. Chen, P. Xia, M. Chen, et al., A Sequential Epitope-Protein Imprinting Strategy for Antibody-Free Electrochemical Sensing of CD44 Glycoprotein with Ultralow Detection Limit, Anal. Chem. (2026). <https://doi.org/10.1021/acs.analchem.5c07439>

[30] A. Yarman, A. Oktay, M. Işık Toksoy, et al., Spotlights of MIP-sensors for drugs and protein biomarkers, J. Pharm. Biomed. Anal. Open 5 (2025) 100048. https://doi.org/10.1016/j.jpbao.2024.100048

[31] W. Wan, H. Zhao, B. Qian, et al., A molecularly imprinted electrochemical sensor based on nitrogen-doped graphene oxide nanoribbons and Au-Pt bimetallic nanoparticles for ultrasensitive detection of perfluorooctane sulfonate (PFOS) in aqueous medium, Microchem. J. 215 (2025) 114256. https://doi.org/10.1016/j.microc.2025.114256

[32] S. Espinoza-Torres, A. Choquehuanca-Azaña, M. Rufino, et al., Development of an Electrochemical Sensor Based on Molecularly Imprinted Polymer Using Functionalized Gold Nanoparticles for Caffeine Quantification, Biosensors 15 (10) (2025) 704. https://doi.org/10.3390/bios15100704

[33] J. Wei, Y. Shen, G. Ouyang, Determination of circulating tumor cells by surface-enhanced Raman scattering based on molecularly imprinted polymers doped with silver nanoparticles, Anal. Sci. 41 (4) (2025) 447–455. https://doi.org/10.1007/s44211-025-00719-7

[34] W. Khaerani, Irkham, U. Pratomo, et al., Platinum-modified screen-printed carbon electrode integrated with molecularly imprinted polymer for highly selective electrochemical ethanol sensing, Electrochem. Commun. 177 (2025) 107986. https://doi.org/10.1016/j.elecom.2025.107986

[35] Y. Zeng, M. Zhang, L. Ding, et al., Molecularly imprinted polymer photoelectrochemical sensor for the detection of triazophos in water based on carbon quantum dot-modified titanium dioxide, Microchim. Acta 191 (5) (2024) 277. https://doi.org/10.1007/s00604-024-06364-7

[36] A. Al Faysal, A. Cetinkaya, T. Erdoğan, et al., Creation of an ultra-sensitive and specific molecularly imprinted polymer sensor with ZnO nanoparticle assistance for valganciclovir detection, Microchim. Acta 193 (1) (2026) 30. https://doi.org/10.1007/s00604-025-07767-w

[37] Archana, Z. Hashmi, H. Bhardwaj, et al., Reduced Graphene Oxide Nanosheets Enhance the Sensitivity of p-Cresyl Sulfate Detection Using a Molecularly Imprinted Polymer-Based Electrochemical Sensor, ACS Appl. Polym. Mater. 6 (11) (2024) 6278–6289. https://doi.org/10.1021/acsapm.4c00435

[38] J. Chuiprasert, S. Srinives, N. Boontanon, et al., Electrochemical sensor based on a composite of reduced graphene oxide and molecularly imprinted copolymer of polyaniline-poly(o-phenylenediamine) for ciprofloxacin determination, ACS Omega 8 (2) (2023) 2564–2574. https://doi.org/10.1021/acsomega.2c07095

[39] T. Cowen, S. Grammatikos, M. Cheffena, Molecularly imprinted polymer nanoparticle-carbon nanotube composite electrochemical gas sensor for highly selective and sensitive detection of methanol vapour, Analyst 149 (8) (2024) 2428–2435. https://doi.org/10.1039/D4AN00045E

[40] S. Mahmodnezhad, M. Roushani, Z.M. Karazan, An electrochemical sensor based on the molecularly imprinted polymer/single walled carbon nanotube-modified glassy carbon electrode for detection of zineb fungicide in food samples, Food Control 168 (2025) 110919. https://doi.org/10.1016/j.foodcont.2024.110919

[41] Y. Xu, P. Zhou, T. Cui, Laser-induced graphene sensors with electropolymerized molecularly imprinted polymer for the detection of ultra-low perfluorooctanoic acid, Sens. Actuators B Chem. 445 (2025) 138635. https://doi.org/10.1016/j.snb.2025.138635

[42] W. Yuan, Y. Wang, M. Jiang, et al., Research on a molecularly imprinted electrochemical sensor based on a graphene quantum dot-gold nanoparticle composite for the determination of 17β-estradiol, Anal. Methods 17 (5) (2025) 999–1009. https://doi.org/10.1039/D4AY01943A

[43] N. Xiang, S. Qi, J. Piao, Development of a molecularly imprinted electrochemical sensor based on (Cu-BTC)-MOF and graphene composite for highly sensitive and selective chloramphenicol detection, RSC Adv. 16 (20) (2026) 17869–17879. https://doi.org/10.1039/D5RA10071B

[44] Y. Zhong, Z. Li, A. Zhang, et al., A molecularly imprinted electrochemical sensor MIP/Cu-MOF/rGO/AuNPs/GCE for highly sensitive detection of electroneutral organophosphorus pesticide residues, Microchim. Acta 191 (6) (2024) 338. https://doi.org/10.1007/s00604-024-06420-2

[45] W. Liu, Z. Wu, J. Peng, et al., Construction of a molecularly imprinted fluorescent sensor based on an amphiphilic block copolymer-metal-organic framework for the detection of oxytetracycline in milk, Anal. Methods 16 (2) (2024) 196–204. https://doi.org/10.1039/D3AY01567J

[46] Y. Qi, Y. Chen, Q. Li, et al., A novel ratiometric electrochemical sensing platform combined with molecularly imprinted polymer and Fe-MOF-NH2/CNTs-NH2/MXene composite for efficient detection of ofloxacin, Anal. Chim. Acta 1316 (2024) 342876. https://doi.org/10.1016/j.aca.2024.342876

[47] B. Li, X. Liao, L. Wang, et al., Molecularly imprinted polymer coated Eu-BCA MOF for highly selective detection of Zearalenone in corn oil, Food Chem. 489 (2025) 144872. https://doi.org/10.1016/j.foodchem.2025.144872

[48] L. Deng, J. Liu, H. Huang, et al., A molecularly imprinted electrochemical sensor based on TiO2@Ti3C2Tx for highly sensitive and selective detection of chlortetracycline, Molecules 28 (22) (2023) 7475. https://doi.org/10.3390/molecules28227475

[49] H. Liu, W. Qin, X. Li, et al., Molecularly imprinted electrochemical sensors based on Ti3C2Tx-MXene and graphene composite modifications for ultrasensitive cortisol detection, Anal. Chem. 95 (44) (2023) 16079–16088. https://doi.org/10.1021/acs.analchem.3c01715

[50] S.N. Samanci, S.I. Kaya, M.A. Unal, et al., Eco-friendly nanoscale recognition: a MIP-based electrochemical sensor for selective determination of abacavir in pharmaceutical and serum samples, Anal. Bioanal. Chem. 418 (2026) 1081–1097. https://doi.org/10.1007/s00216-025-06261-9

[51] J.A. Cabas Rodríguez, F.J. Arévalo, A.M. Granero, Design of an MIP-Based Electrochemical Sensor for the Determination of Paracetamol in Pharmaceutical Samples, Biosensors 15 (8) (2025) 544. <https://doi.org/10.3390/bios15080544>

[52]	S. Sardaremelli, M. Hasanzadeh, H. Razmi, Chemical binding of horseradish peroxidase enzyme with poly beta‐cyclodextrin and its application as molecularly imprinted polymer for the monitoring of <scp> H 2 O 2 </scp> in human plasma samples, J. Mol. Recognit. 34 (5) (2021). https://doi.org/10.1002/jmr.2884

[53]	M.A. Samy, M.A.-H. Abdel-Tawab, N.T. Abdel-Ghani, et al., Application of Molecularly Imprinted Microelectrode as a Promising Point-of-Care Biosensor for Alanine Aminotransferase Enzyme, Chemosensors 11 (5) (2023) 262. https://doi.org/10.3390/chemosensors11050262

[54]	T.S. Rebelo, I.M. Miranda, A.T. Brandão, et al., A disposable saliva electrochemical MIP-based biosensor for detection of the stress biomarker α-amylase in point-of-care applications, Electrochem 2 (3) (2021) 427–438. https://doi.org/10.3390/electrochem2030028

[55]	T. Di Giulio, E. Mazzotta, C. Malitesta, Molecularly Imprinted Polyscopoletin for the Electrochemical Detection of the Chronic Disease Marker Lysozyme, Biosensors 11 (1) (2020) 3. https://doi.org/10.3390/bios11010003

[56]	P. Montoro-Leal, I.A.M. Frías, E. Vereda Alonso, et al., A Molecularly Imprinted Polypyrrole/GO@Fe3O4 Nanocomposite Modified Impedimetric Sensor for the Routine Monitoring of Lysozyme, Biosensors 12 (9) (2022) 727. https://doi.org/10.3390/bios12090727

[57]	P. Singla, S. Kaur, O. Jamieson, et al., Electrochemical and thermal detection of allergenic substance lysozyme with molecularly imprinted nanoparticles, Anal. Bioanal. Chem. 415 (18) (2023) 4467–4478. https://doi.org/10.1007/s00216-023-04638-2

[58]	D.Y. Choi, J.C. Yang, J. Park, Optimization and characterization of electrochemical protein Imprinting on hemispherical porous gold patterns for the detection of trypsin, Sens. Actuators B Chem. 350 (2022) 130855. https://doi.org/10.1016/j.snb.2021.130855

[59] S. Di Masi, M. Costa, F. Canfarotta, et al., An impedimetric sensor based on molecularly imprinted nanoparticles for the determination of trypsin in artificial matrices–towards point-of-care diagnostics, Anal. Methods 16 (5) (2024) 742–750. https://doi.org/10.1039/D3AY01762A

[60]	K. Guo, Fabrication of a molecular imprinting electrochemical sensor for detecting acid phosphatase in black bean sprouts, J. Food Meas. Charact. 18 (1) (2024) 640–646. https://doi.org/10.1007/s11694-023-02217-8

[61]	S. Yeasmin, A. Ullah, B. Wu, et al., Enzyme-Mimics for Sensitive and Selective Steroid Metabolite Detection, ACS Appl. Mater. Interfaces (2023). https://doi.org/10.1021/acsami.2c21980

[62]	W. Lian, X. Zhang, Y. Han, et al., A Molecularly Imprinted Electrochemical Sensor for Carbendazim Detection Based on Synergy Amplified Effect of Bioelectrocatalysis and Nanocomposites, Polymers 17 (1) (2025) 92. https://doi.org/10.3390/polym17010092

[63] H. Özçelik, E. Sezer, E. Dokuzparmak, et al., Beyond linear calibration: A split-and-conquer AI architecture for MIP-based electrochemical sensing of C-reactive protein, Microchem. J. 225 (2026) 117983. <https://doi.org/10.1016/j.microc.2026.117983>

[64] S. Dokuzparmak, E. Sezer, E. Yaşar, et al., Towards a Scalable, AI-Augmented Purification Platform for Amphetamine-Type Stimulants: From Nanopolymer Synthesis to Predictive Process Simulation, Polym. Adv. Technol. 37 (5) (2026) e70627.

[65] E. Sezer, F.U. Ulucan Karnak, S. Akgöl, Green carbon dots in the era of AI: sustainable synthesis, intelligent drug delivery, advanced diagnostics, and bioimaging, Turkish J. Biol. 49 (5) (2025) 498–533. <https://doi.org/10.55730/1300-0152.2762>

[66] E. Sezer, E. Dokuzparmak, H. Özçelik, et al., Harnessing machine learning to revolutionize electrochemical detection of vitamin E acetate in e-liquids, ACS Omega 10 (25) (2025) 27098–27111. https://doi.org/10.1021/acsomega.5c02363

[67] B. Yarahmadi, S.M. Hashemianzadeh, S.M.-R. Milani Hosseini, Machine-learning-based predictions of imprinting quality using ensemble and non-linear regression algorithms, Sci. Rep. 13 (1) (2023) 12111. https://doi.org/10.1038/s41598-023-39374-1

[68] S. Rajpal, P. Mishra, B. Mizaikoff, Rational In Silico Design of Molecularly Imprinted Polymers: Current Challenges and Future Potential, Int. J. Mol. Sci. 24 (7) (2023) 6785. <https://doi.org/10.3390/ijms24076785>

[69] I. Cooley, W. Wang, V. Kozyrev, et al., Predictive Approaches for <scp>3D</scp>‐Printing: Methods and Approaches for Polymeric Materials, WIREs Comput. Mol. Sci. 15 (5) (2025). https://doi.org/10.1002/wcms.70048

[70] W.T. Guo, A. Zahran, H. Ibrahim, et al., Multimodal biosensing platforms for sensor-computing integration towards intelligent healthcare, Soft Sci. 6 (2) (2026). <https://doi.org/10.20517/ss.2025.108>

[71] Y. Liu, X. Liu, X. Wang, et al., AI-Empowered Electrochemical Sensors for Biomedical Applications: Technological Advances and Future Challenges, Biosensors 15 (8) (2025) 487. <https://doi.org/10.3390/bios15080487>

[72] S. Ghosh, R. Bhuvanakantham, P. Sindhujaa, et al., A Cloud-Aware Scalable Architecture for Distributed Edge-Enabled BCI Biosensor System, Biosensors 16 (3) (2026) 157. <https://doi.org/10.3390/bios16030157>
